# Abrupt transition to irreversible damage in the overdrafted Sacramento Valley aquifer system

**DOI:** 10.1073/pnas.2526041123

**Published:** 2026-07-27

**Authors:** Stacy Larochelle, Kristel Chanard, Manon Dalaison, Jérôme Fortin, Romain Jolivet, Laurent Longuevergne, Luce Fleitout, Donald F. Argus, Louis-Marie Gauer, Jean-Philippe Avouac

**Affiliations:** ^a^ Department of Earth, Planetary, and Space Sciences, University of California Los Angeles, Los Angeles, CA 90095; ^b^ Division of Geological and Planetary Sciences, California Institute of Technology, Pasadena, CA 91125; ^c^ Lamont-Doherty Earth Observatory, Columbia University, Palisades, NY 10964; ^d^ Université Paris Cité, Institut de physique du globe de Paris, CNRS, Institut National de l’information géographique et forestière, Paris 75005, France; ^e^ Laboratoire de Géologie, École Normale Supérieure, CNRS, Université Paris Sciences et Lettres, Paris 75005, France; ^f^ Géosciences Rennes, Univ Rennes, CNRS, Rennes 35000, France; ^g^ Jet Propulsion Laboratory, California Institute of Technology, Pasadena, CA 91109

**Keywords:** groundwater, subsidence, drought, geodesy

## Abstract

Groundwater extraction decreases water pressure in aquifer systems, causing reversible or irreversible deformation of the water-bearing layers that manifests as recoverable or permanent displacements of the land surface, respectively. Detecting and forecasting when and where an aquifer system transitions from a reversible, poroelastic regime, to an irreversible, inelastic regime remains a crucial challenge given the complex, heterogeneous nature of aquifer systems. Here we leverage high-resolution measurements of ground deformation and groundwater levels from 2016 to 2022 to characterize both regimes at the regional scale and show that a critical transition occurred in large areas of the Sacramento Valley during California’s 2020–2022 extreme drought. Our analysis reveals that, while deformation remained primarily poroelastic during the 2016–2020 interdrought period, land subsidence in areas of intense groundwater extraction accelerated abruptly in 2021, with subsidence rates exceeding the inferred poroelastic rates by several decimeters per year. Such rapid and extensive land subsidence indicates severe inelastic compaction and loss of storage capacity of the underlying aquifer system, which pose a serious threat to California’s water resources and infrastructure. A comparison of present-day deformation with historical groundwater levels reveals that this abrupt transition was not predictable based on the available groundwater records alone.

## Significance

Groundwater extraction can cause both reversible and irreversible ground deformation, with the latter leading to permanent land subsidence and loss of groundwater storage capacity. Satellite observations of land surface deformation reveal that large areas of the Sacramento Valley abruptly transitioned from reversible to irreversible compaction over the 2020–2022 California drought—a transition not anticipated from the groundwater monitoring network alone. Subsidence rates reaching several decimeters per year and not following groundwater levels indicate lasting damage to the aquifer system and underscore the need for more conservative groundwater management. By imaging this sharp transition at the regional scale, our analysis demonstrates the potential of space-based monitoring for early detection of groundwater overdraft and the resulting loss of aquifer storage capacity worldwide.

**Fig. 1.**
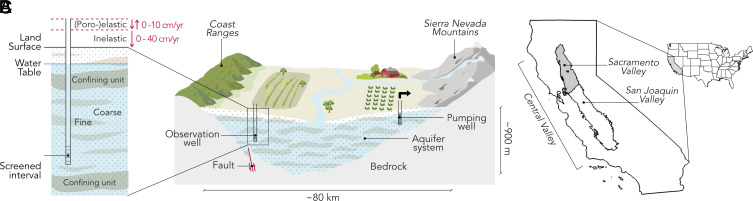
Hydrogeological setting of the Sacramento Valley. (*A*) Schematic diagram of groundwater level changes and resulting poroelastic and inelastic land surface deformation [adapted from Galloway et al. (3)]. (*B*) Idealized hydrogeological cross-section of the Sacramento Valley aquifer system [adapted from Faunt et al. (29)]. (*C*) Location of the Sacramento Valley and San Joaquin Valley, together making up California’s Central Valley.

As global demand for groundwater intensifies in response to climate change, its ability to act as a buffer against surface water variability becomes increasingly critical (1). A growing concern, however, is that intensive exploitation of groundwater resources can damage aquifer systems and hence threaten future groundwater availability (2). Removing water stored in the pores of an aquifer system stresses its solid matrix since pressurized water helps support the weight of overlying materials (3). The system can respond to this stress perturbation either elastically or inelastically. In the poroelastic regime, water-bearing pores deform elastically: They contract as fluid pressure decreases during net groundwater discharge and expand when fluid pressure increases during recharge (4), causing no permanent damage to the porous rock matrix. Current theories imply that when fluid pressure reaches a historic low during intensive groundwater extraction, the system might enter the inelastic regime in which pores permanently contract, leading to irreversible compaction, reduction of storage capacity and permeability that may impede aquifer recharge (5, 6). While poroelastic deformation results in reversible displacements of the Earth’s surface, inelastic deformation leads to permanent land subsidence (Fig. 1*A*). Measurements of surface deformation over exploited aquifer systems provide a way to monitor groundwater management practices and mitigate risks including infrastructure damage, increased flood vulnerability and other socioeconomic consequences of land subsidence (7).

Over the last two decades, space-based geodetic techniques like Global Navigation Satellite Systems (GNSS) and Interferometric Synthetic Aperture Radar (InSAR) have revolutionized aquifer monitoring by enabling the measurement of surface deformation with unprecedented spatial coverage, decametric resolution, and millimeter/y accuracy. Measurements of surface deformation provide depth-integrated insight into the complex mechanical response of heterogeneous aquifer systems to internal pressure changes (Fig. 1*A*). These observations complement basin-scale estimates of groundwater mass changes (8, 9) inferred from the Gravity Recovery and Climate Experiment and Follow-On (GRACE/-FO) satellite missions (10, 11), as well as local-scale in-situ hydrogeophysical (12, 13) and hydrological monitoring (14). Geodetic techniques have been used to document both poroelastic (15–20) and inelastic (6, 21–25) deformation in diverse aquifer settings. Yet, identifying when and where aquifer systems transition from one regime to another remains challenging, in part due to the inherently heterogeneous nature of these systems as well as to the limited availability of temporally and spatially coincident measurements of surface deformation and water pressure at depth. Extracting poroelastic and inelastic deformation signals from noisy geodetic datasets that can contain other sources of deformation (e.g., tectonic, hydrological and atmospheric loading, and thermal effects) also remains challenging (20, 26, 27). Together, these factors limit our ability to identify, forecast, and prevent the onset of damaging inelastic compaction.

Here, we leverage InSAR and GNSS measurements of land-surface displacements, GRACE/-FO observations of large-scale water-mass changes, and in situ well measurements of groundwater levels to characterize the poroelastic and inelastic deformation regimes in the Sacramento Valley during the 2016–2020 interdrought period and the 2020–2022 California-wide drought. Our regional-scale analysis reveals the abrupt and widespread transition of the aquifer system to the damaging inelastic regime in 2021—an event not predictable from the available historical groundwater level records, despite the underlying mechanics of such transitions being well established in theory.

## Hydrogeologic Setting and History of Land Subsidence

The Sacramento Valley makes up the northern one-third of the Central Valley sedimentary basin nestled between the Coast Ranges and Sierra Nevada mountains (Fig. 1 *B* and *C*). The southern two-third of the Central Valley is known as the San Joaquin Valley (Fig. 1*C*). The continental deposits that form the Sacramento Valley average 350 m in thickness and can reach up to 920 m in the southwest part of the valley (28). While the San Joaquin Valley features a distinctive continuous Corcoran clay layer, the Sacramento Valley is characterized by discontinuous clay lenses (29) (Fig. 1 *A* and *B*). Fine-grained sediments like clays and silts are abundant throughout the Sacramento Valley, making up an estimated 64% of the upper 400 m of the aquifer system, and are especially prevalent west of the Sacramento River (30) (Lithology in *Materials and Methods*). Altogether, these deposits can be conceptualized as a single aquifer unit with an impermeable bedrock basement and spatially heterogeneous confinement and hydraulic properties (28–30). In addition to direct precipitation, a significant portion of aquifer recharge originates from precipitation and meltwater from the neighboring Sierra Nevada mountains. This meltwater is channeled through a network of dammed reservoirs, rivers, engineered canals, and irrigation systems, some of which ultimately reaches the aquifer below.

**Fig. 2.**
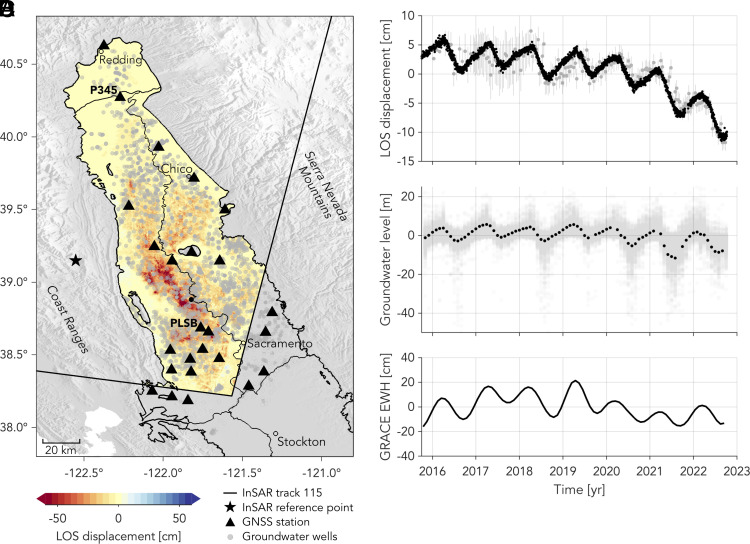
Multitechnique observations of the Sacramento Valley aquifer system in 2016–2022. (*A*) Cumulative LOS InSAR displacement from Sept. 2016 to Sept. 2022 (colormap), referenced to a stable point in the Coast Ranges, west of the valley (star). Locations of the 25 GNSS stations (triangles) and 2,535 groundwater wells (circles). (*B*) LOS InSAR (gray) and GNSS (black) displacements at GNSS station PLSB, referenced to GNSS station P345, the locations of which are indicated in (*A*). (*C*) Stacked (gray) and sample (black) groundwater level deviations from their mean depth. The location of the sample time series is shown in (*A*) (black circle). (*D*) Mean GRACE equivalent water height (averaged over 38° to 41° latitude and $-123^{{}^{\circ}}$ to $-121^{{}^{\circ}}$ longitude box).

The Central Valley has a century-long history of groundwater exploitation to sustain its intensive agriculture economy, which produces roughly a quarter of the United States food supply—primarily from water-intensive crops such as rice, fruits, and nuts (29, 31). This dependence is evidenced by the thousands of groundwater wells distributed across the region (Fig. 2*A*). In the San Joaquin Valley, sustained overdraft has led to well-documented, widespread inelastic deformation since the mid-1920s (32), including as much as 9 m of subsidence recorded by leveling near Mendota in the center of the San Joaquin Valley, between 1925 and 1977 (33). InSAR observations further revealed large-scale ongoing subsidence in the region, with rates reaching several decimeters per year (6, 26, 34–36). In contrast, observations of permanent land subsidence in the Sacramento Valley have so far been relatively limited and of smaller amplitudes (29). Leveling surveys suggest that localized areas in the southwestern Sacramento Valley experienced land subsidence on the order of 0.5 m between 1935 and 1964 (37). Similar subsidence rates persisted through the 1990s, as evidenced by additional leveling data, GPS surveys, extensometers, and reports of failed well casings, with increased rates during drought periods (7, 29, 38). The few InSAR-based studies covering the Sacramento Valley only report relatively small subsidence rates during the 2007-2010 drought (6, 39) and over the 2015–2019 period (27). More recently, extensive campaign GPS surveys conducted in 2008 and 2017 revealed significant cumulative subsidence reaching up to 0.65 m, most of which likely occurred during the 2012–2016 drought (40). In view of the long-standing recognition of groundwater depletion and associated land subsidence in the Central Valley, the California Sustainable Groundwater Management Act (SGMA) (31) enacted a statewide regulation in 2014. In particular, SGMA designated many subbasins in the San Joaquin Valley as “critically overdrafted”, but none in the Sacramento Valley.

## Multitechnique Observations in 2016–2022

Over the 2016–2022 period considered here, InSAR measurements reveal large, widespread Line-Of-Sight (LOS) deformation, with amplitudes reaching several decimeters (Fig. 2*A*; InSAR in *Materials and Methods*; *SI Appendix*, section S1). We validate these InSAR measurements against GNSS observations (GNSS in *Materials and Methods*), both referenced to station P345 in the northern Sacramento Valley (Fig. 2*A*). The two datasets generally show good agreement (e.g., Fig. 2*B*), both within the valley and at surrounding stable sites (*SI Appendix*, section S1). However, none of the GNSS stations are located in the area of most rapid deformation identified with InSAR. This likely reflects the deployment strategy of permanent GNSS networks, which were originally designed to study tectonic processes and are therefore preferentially installed on relatively stable ground (41). In contrast, our InSAR analysis provides dense spatial coverage, including agricultural and natural terrains prone to decorrelation (e.g., over grasslands), through a small-baseline subset (SBAS) approach combined with a Kalman-filter time-series analysis to robustly estimate displacements and associated uncertainties (42).

The large-amplitude seasonal signals observed in the surface displacement time series (Fig. 2*B*) primarily reflect pore-water pressure changes within the aquifer system. Indeed, stacking groundwater-level measurements, relative to their mean depth, from the ∼2,500 available wells from 2016 to 2022 (Groundwater levels in *Materials and Methods*) reveals large-amplitude seasonal fluctuations in pore-water pressure (Fig. 2*C*), which reflect cycles of groundwater extraction during the summer and net aquifer recharge during winter and spring (29). This temporal behavior is consistent with the larger-scale changes in total water mass inferred from GRACE/-FO observations, at a spatial resolution of a few hundreds kilometers (10, 11, 43) (Fig. 2*D*; GRACE/-FO in *Materials and Methods*). Both groundwater levels and GRACE-measured total water mass also exhibit a multiannual pattern associated with recovery from the 2012–2016 drought and onset of the 2020–2022 drought (Fig. 2 *C* and *D*).

## Groundwater Fluctuations and Long-Term Decline

To characterize groundwater-level fluctuations relevant to aquifer-scale deformation, we construct a representative regional groundwater signal by averaging all detrended groundwater time series and projecting each individual time series onto this mean signal (Groundwater projections in *Materials and Methods*; *SI Appendix*, section S2). This approach uses the observed mean groundwater time series to effectively capture the dominant temporal variations shared across most wells, including seasonal fluctuations and multiannual drought signals, as well as the asymmetric and nonperiodic characteristics of the dataset. While the mean time series explains 51% of the dataset variance and does not reproduce all local variability, it provides a first-order representation of regional groundwater fluctuations, mitigates the limitations of sparse individual records, and enables comparison with geodetic observations. We estimate groundwater-level trends from 2016 to 2022 by performing a linear fit through the residual groundwater level time series after subtracting the contribution from the mean time series.

**Fig. 3.**
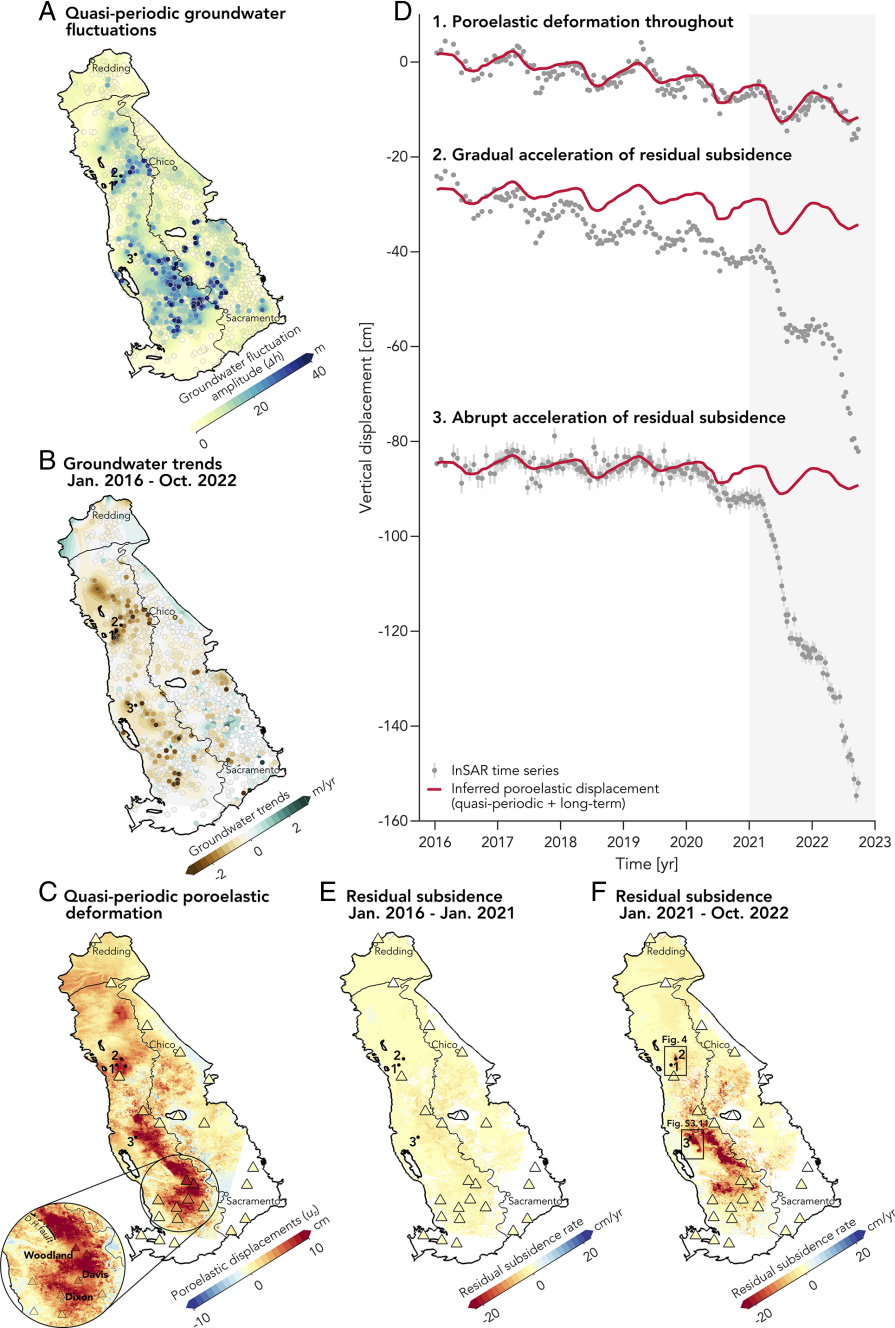
Imaging the abrupt transition from predominantly poroelastic to predominantly inelastic deformation in 2021. Spatial distributions of (*A*) groundwater level fluctuations, (*B*) groundwater level trends from January 2016 to October 2022, (*C*) vertical poroelastic deformation measured by InSAR (colormap) and GNSS (triangles) at the seasonal and drought timescales. Spatial distributions of residual subsidence rates during (*D*) January. The *Inset* in (*C*) provides a closer look at urban areas and the Dunnigan Hills (D.H.) fault. Groundwater fluctuations and poroelastic deformation correspond to the (peak-topeak) projection amplitudes. (*D*) InSAR displacements (gray dots) and inferred poroelastic displacements (red line) at the sample points indicated in the other panels, illustrating the 3 characteristic behaviors. The gray shaded area indicates the period of accelerated subsidence in 2021–2022. Spatial distributions of residual subsidence rates during (*E*) January 2016–2021 and (*F*) January 2021 to October 2022, obtained by subtracting the seasonal and long-term poroelastic deformation [red curves in (*D*)] from the observations [gray dots in (*D*)].

Fig. 3 *A* and *B* shows the resulting spatial distributions of seasonal amplitudes (as determined by the projection) and long-term rates, with each well sampling a different depth interval with different sampling frequency. The interpolated maps in the background reflect effective values across all sampled aquifer depths. Fig. 3*A* reveals widespread zones of high-amplitude oscillations west of the Sacramento River, with fluctuations up to 40 m, consistent with heavy pumping activity in these areas (9, 44). In contrast, long-term groundwater rates are more spatially localized than the quasi-periodic fluctuations, with decline rates exceeding −2 m/y confined to a few areas, primarily in the northwest portion of the valley (Fig. 3*B*).

## Extracting Poroelastic and Inelastic Deformation

To characterize the poroelastic and inelastic deformation regimes over the study period, we first isolate the poroelastic contribution from the InSAR and GNSS datasets by 1) removing the elastic loading response of the solid Earth to changes in total water mass (45–47), 2) extracting the part of residual displacements that correlates with groundwater-level fluctuations, and 3) estimating long-term poroelastic displacements from the observed groundwater trends.

Starting from the detrended vertical displacement time series (InSAR and GNSS in *Materials and Methods*), we estimate and remove the elastic loading response associated with global mass redistribution measured by GRACE/-FO (48) (GRACE/-FO deformation model in *Materials and Methods*; *SI Appendix*, section S1). Since the InSAR time series are referenced to a stable point west of the valley (Fig. 2*A*), we correct the InSAR data for spatial gradients in the modeled elastic deformation field relative to this reference, which primarily reflect the influence of the nearby Sierra Nevada Mountains. Because GRACE/-FO captures only large-scale (∼300 to 400 km) hydrological mass changes, the residual InSAR and GNSS time series may contain deformation from smaller-scale hydrological loads (27). However, as elastic loading displacements scale with load extent (20), we assume that the GRACE/-FO correction is sufficient for isolating centimeter-scale poroelastic and inelastic deformation of the aquifer.

We then project the residual, detrended geodetic time series onto the mean groundwater time series (20) (Groundwater projections in *Materials and Methods*). This approach helps reduce local and regional effects that may be due to other geophysical processes (e.g., imperfect elastic loading corrections, thermoelastic, or tectonic deformation) or systematic technique errors (e.g., GNSS multipath, residual atmospheric delays, orbit modeling deficiencies, or InSAR closure biases), which can reach up to a few centimeters in both GNSS and InSAR time series (42, 49–51). To ensure the recovered deformation signal is predominantly poroelastic, we limit the projection interval to the Jan. 2016 to Dec. 2020 interdrought period, during which groundwater levels are generally recovering from the 2012–2016 drought (Fig. 2*C*) and, hence, no significant instantaneous inelastic deformation is expected at the aquifer scale. This approach may underestimate poroelastic deformation where local groundwater variations depart from the mean signal. However, a more precise assessment of localized deformation would require site-specific analyses, which are often limited by incomplete groundwater records.

We estimate poroelastic deformation driven by long-term groundwater trends by multiplying the observed groundwater trends (Fig. 3*B*) by the ratio of quasi-periodic poroelastic deformation ($u_{z}$; Fig. 3*C*) to groundwater fluctuations (Δh; Fig. 3*B*), where $u_{z}/\Delta h$ quantifies the depth-integrated elastic compressibility of the aquifer system (*SI Appendix*, section S3). We note that this compressibility may differ between seasonal and decadal timescales due to pressure- and temperature-dependent effects, but by at most a few percents (52). Moreover, the approach should remain approximately valid even if some units deform inelastically, as other units within the aquifer column are still expected to respond poroelastically. While the inferred deformation relies on the ability of the available groundwater dataset to adequately capture trends in the deforming units, this framework nonetheless provides a first-order estimate of long-term poroelastic deformation at the aquifer scale—an effect that is often overlooked.

Finally, we assume that the residual displacements, after removing the modeled elastic loading and inferred poroelastic components (i.e., the sum of the quasi-periodic (Fig. 3*C*) and long-term (*SI Appendix*, Fig. S3.2) signals), primarily reflect inelastic deformation.

## Primarily Poroelastic Regime During the 2016–2020 Interdrought Period

Our analysis reveals large-amplitude quasi-periodic poroelastic displacements concentrated in areas of large groundwater fluctuations (Fig. 3 *A* and *C*), with amplitudes independently obtained from InSAR and GNSS agreeing within 2.5 cm (*SI Appendix*, section S3). Zooming in on one of the zones with large poroelastic fluctuations (*Inset* in Fig. 3*A*) reveals smaller displacements around urban areas like Woodland, Davis, and Dixon, and larger displacements in the surrounding agricultural lands where irrigation occurs. The *Inset* also reveals a sharp discontinuity in displacement amplitudes along the Dunnigan Hills fault line, suggesting that groundwater flow across the fault is restricted, as concluded from similar observations in other aquifer systems (23, 53, 54).

Significant long-term poroelastic subsidence rates, reaching up to 2 cm/y, also occur in regions experiencing substantial groundwater decline (*SI Appendix*, Fig. S3.2 and Fig. 3*B*), suggesting that a nonnegligible portion of long-term subsidence during this period is poroelastic and therefore reversible.

We find that, together, these poroelastic signals explain most of the observed surface displacements across the valley over the 2016–2020 interdrought period (Fig. 3*D* and *SI Appendix*, section S3). This agreement suggests that 1) the mean groundwater time series captures the dominant groundwater fluctuations driving aquifer-scale deformation and 2) inelastic deformation was relatively small during the 2016–2020 interdrought period, as evidenced by the generally small residual subsidence rates from January 2016 to 2020 (Fig. 3*E*). A few locations do show significant residual trends over this period (e.g., Point 2 in Fig. 3*D*), which may reflect delayed inelastic compaction due to extraction prior to 2016, inaccuracies in the estimated long-term poroelastic signal due to the methodological limitations discussed above, or other nonhydrological deformation processes.

## Abrupt and Widespread Acceleration of Subsidence in 2021

Starting in 2021, large areas of the valley undergo rapid subsidence, significantly deviating from the inferred poroelastic deformation (Fig. 3 *D* and *F*). Subsidence rates from Jan. 2021 to Oct. 2022 reach up to 50 cm/y in some localized areas, comparable to those observed in the critically overdrafted San Joaquin Valley (6), and far exceed the InSAR velocity uncertainty, which is below 2 cm/y (*SI Appendix*, section S1). Comparing the average rates of residual deformation across the valley between 2016 and 2021 (Fig. 3*D*) and 2021–2022 (Fig. 3*E*) reveals the dramatic and widespread nature of this accelerated subsidence. The zones of rapid subsidence identified here largely coincide with areas that experienced significant subsidence between 2008 and 2017, as documented by a campaign GPS survey (40) (*SI Appendix*, Fig. S3.7).

All available measurements suggest that the accelerated subsidence starting in 2021 primarily results from inelastic compaction due to groundwater extraction. The rapidly subsiding areas coincide with zones of intensive pumping identified in the groundwater fluctuations and poroelastic deformation maps (Fig. 3 *A* and *C*). Moreover, comparison of groundwater levels and InSAR displacements in the zones of accelerated subsidence shows that, on average, the ground surface does not rebound to its pre-2021 state even as groundwater levels recover with seasonal recharge at the end of 2021 (*SI Appendix*, Fig. S3.8), indicative of irreversible deformation. Groundwater time series do not show a clear change in slope in 2021, suggesting that the abrupt subsidence acceleration cannot be explained by large, localized groundwater deviations from the mean aquifer-scale behavior. Overall, the magnitude, timing, and spatial extent of the observed deformation strongly support a transition to inelastic compaction as the dominant deformation mechanism during 2021–2022, in contrast to the poroelastic-dominated 2016–2020 interdrought period.

## An Unanticipated Transition in Deformation Regime

**Fig. 4.**
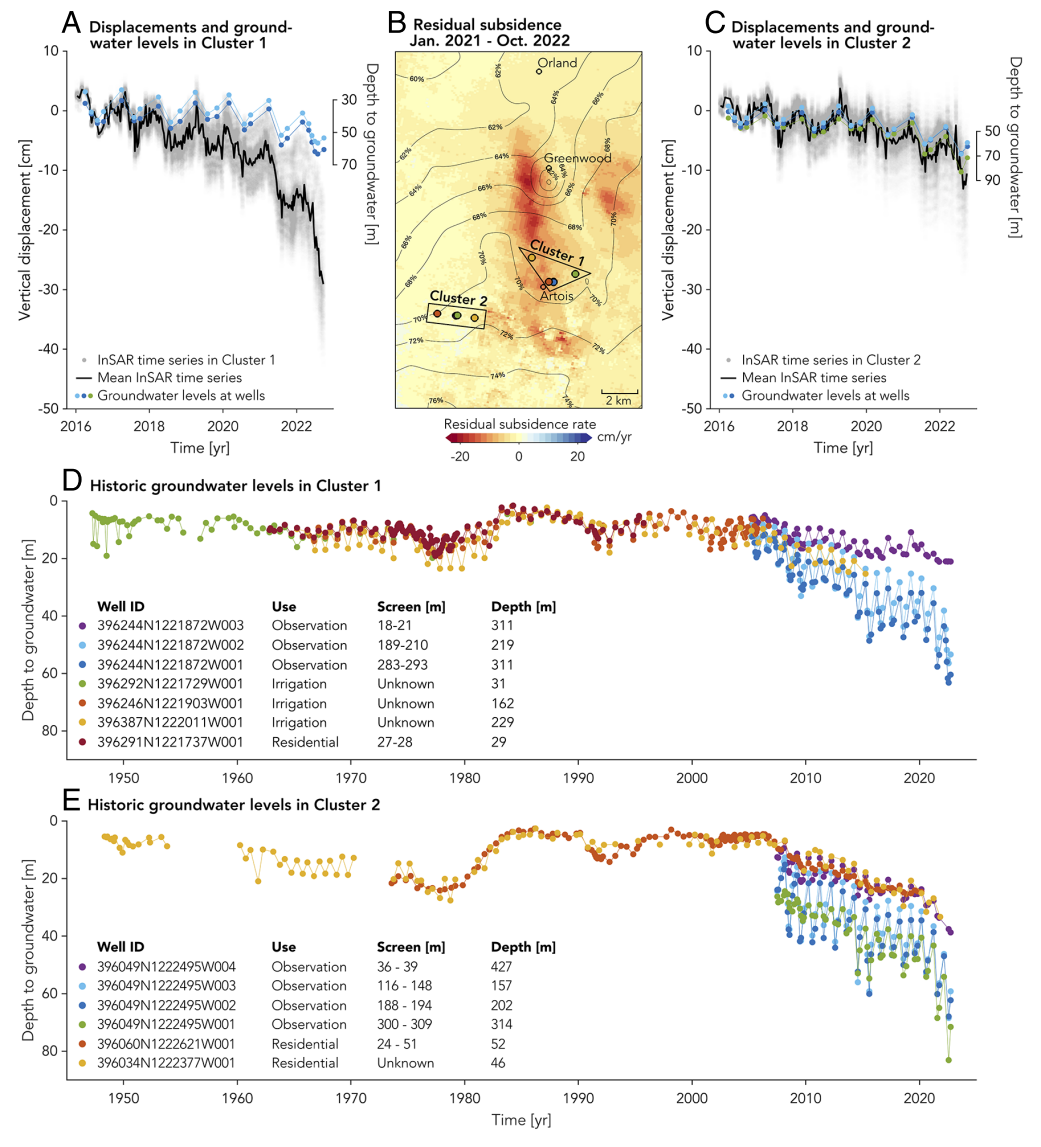
Linking modern deformation to historic groundwater levels. (*A*) Stacked (gray dots) and mean displacements (black curve) at InSAR pixels in Cluster 1. Groundwater levels recorded at historic wells from Jan. 2016 to Oct. 2022, scaled to fit the seasonal deformation, are shown with color dots. (*B*) Residual subsidence rate (background colormap) from Jan. 2021 to Oct. 2022 for the area shown in Fig. 3*E*, percentage of fine-grained materials (contours) and locations of the 2 clusters (black outlines) and historic wells (colored circles). Note that some wells have the same position at the surface but sample different depths. (*C*) Similar to (*A*) but for Cluster 2. (*D* and *E*) Historic groundwater levels recorded at wells within each cluster. The color-coded groundwater levels in (*A* and *C*) are the same as those in (*D* and *E*).

**Fig. 5.**
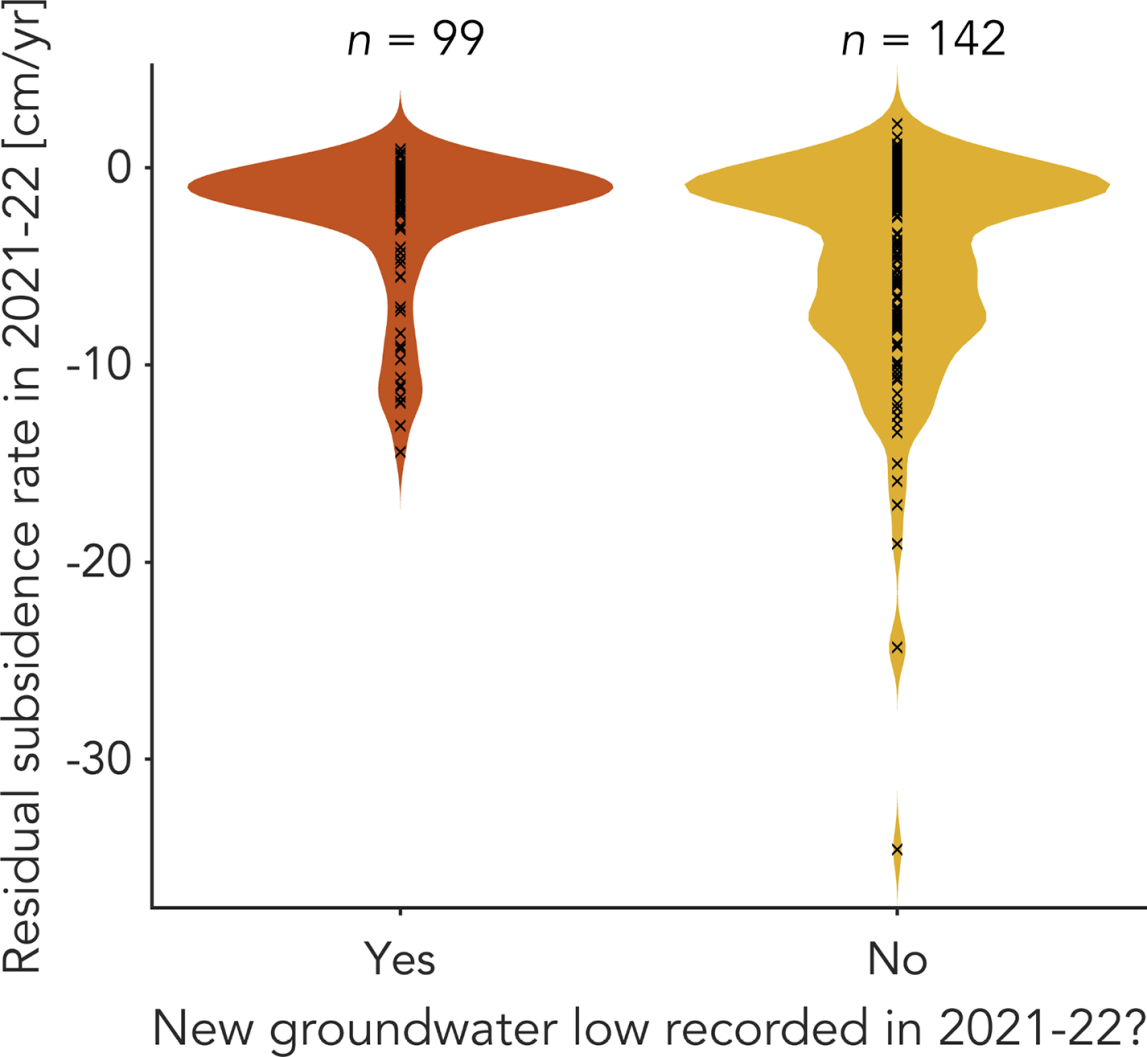
Weak correspondence between subsidence rates and available groundwater records. Distribution of the 2021–2022 residual deformation rates at historic wells that did (“Yes”) and did not (“No”) record a new groundwater level minimum during this period, shown as violin plots.

Inelastic compaction of unconsolidated aquifer systems typically occurs when water pressure within a sedimentary unit reaches a historic low, causing unprecedented stress (the so-called preconsolidation stress) on the porous solid skeleton (3). Cluster 1 exemplifies this behavior, showing both accelerated residual subsidence in 2021–2022 (Fig. 4*A*) and a new recorded groundwater low in 2021 (Fig. 4*D*; Groundwater levels in *Materials and Methods*). However, our datasets show that inelastic deformation often does not coincide with a new historic low in the available groundwater record, and likewise, that new groundwater lows recorded in 2021–2022 are not necessarily associated with significant inelastic deformation. For example, nearby Cluster 2, characterized by a similar fine-grained fraction to Cluster 1 (68 to 72%; Fig. 4*B*) (30), also reached a new recorded groundwater low in 2021, and again in 2022 (Fig. 4*E*) yet continued to deform poroelastically throughout 2021–2022 (Fig. 4*C*). Conversely, other clusters display large residual subsidence rates in 2021–2022, but no recorded groundwater low (*SI Appendix*, section S3). In fact, when considering 241 relatively complete groundwater records at individual wells across the Sacramento Valley (Groundwater levels in *Materials and Methods*; *SI Appendix*, section S2), we find that the majority (70%) of wells that did record a new groundwater low in 2021–2022 are not associated with significant residual subsidence faster than −2 cm/y (Fig. 5). Conversely, more than half (54%) of wells that did not record a new groundwater low show residual subsidence faster than −2 cm/y (Fig. 5). We thus conclude that the 2021 transition from predominantly poroelastic to predominantly inelastic deformation could not have been anticipated from the available groundwater records alone.

## Complex, Heterogeneous Distributions of Water Pressure and Preconsolidation Stress

The weak correspondence between the observed subsidence rates and recorded groundwater lows likely stems from the limited capacity of wells, which measure water pressure only within their screened intervals (Fig. 1*A*) and may miss key epochs of groundwater lows, to reflect conditions in the units undergoing inelastic compaction. After a century-long history of groundwater extraction and natural recharge, complex interactions between the coarser-grained, higher-permeability aquifer units and the finer-grained, lower-permeability interbeds, confining units, and aquitards likely result in heterogeneous distributions of both water pressure and preconsolidation stress in the different units (Fig. 1 *A* and *B*) (3, 7, 55, 56). During groundwater extraction, water is first drawn from the aquifer units, the most permeable interbeds, and the outer edges of thicker confining units. Over time, declining water pressures in the higher-permeability units create a vertical head gradient that causes water to be increasingly drawn from the lower-permeability units, with water pressure generally remaining higher in these lower-permeability units. Water pressure re-equilibration across these various units may take years to centuries due to the slow drainage of pore water through low-permeability, fine-grained materials (7).

As a result, neither the 2021–2022 water pressure nor the preconsolidation stresses of the relevant units are necessarily well characterized by the available groundwater records. For example, the relatively shallow historic wells in Cluster 2 (orange and yellow dots in Fig. 4*E*) likely failed to capture the lowest groundwater levels across the full aquifer column during 1977–1981, as deeper wells show consistently lower levels than shallower wells in 2006–2022. The preconsolidation stress in the deforming units may therefore be substantially higher than that inferred from the available shallow groundwater records. Similarly, the relatively shallow wells in Cluster 3 likely do not reflect the groundwater levels in deeper units, for which there are no measurements from 2016 to 2022 (*SI Appendix*, Fig. S3.9*D*). Moreover, since water pressure generally remains higher in fine-grained units, we expect lower preconsolidation stress in the fine-grained units compared to the coarser-grained units, such that groundwater levels above the coarse-grained units preconsolidation stress can still cause inelastic deformation (57). These examples clearly demonstrate that the available groundwater records alone are insufficient to accurately identify or forecast the abrupt, widespread acceleration of inelastic compaction observed here.

## A Shift in Groundwater Extraction from Coarse- to Fine-Grained Units

The close agreement between surface deformation and the mean groundwater time series during the 2016–2020 interdrought period suggests that the current well network does adequately capture groundwater variations over seasonal to drought timescales. Since irrigation wells are typically screened in the most productive zones of an aquifer system, the groundwater dataset is likely skewed toward pressure variations occurring in the coarser-grained, high-permeability aquifer units and the outer edges of fine-grained formations. The in-phase poroelastic response during this period thus suggests that deformation predominantly occurs within these units. In this context, the abrupt acceleration of subsidence in 2021 may indicate a transition from water being primarily drawn from the coarse-grained layers and the edges of adjacent units to significant extraction from the deeper portions of fine-grained units due to higher vertical head gradients associated with increased extraction and decreased recharge during drought periods. Fine-grained sediments necessary for this transition are abundant throughout the valley, especially in areas of rapid subsidence (29, 30). While inelastic compaction of highly compressible fine-grained sediments likely dominates the subsidence signal (55, 58), coarser-grained aquifer units could slightly contribute to inelastic compaction under severe stress conditions (59, 60).

## Implications for Groundwater Storage

Our high-resolutions maps of groundwater fluctuations (Δh; Fig. 3*A*) and poroelastic displacements ($u_{z}$; Fig. 3*C*) constrain the elastic storage capacity of the aquifer system, specifically the uniaxial elastic storage coefficient, S, which scales with $u_{z}/\Delta h$ through a factor determined by the relative compressibility of the porous matrix, solid grains, and pore water (Elastic storage capacity in *Materials and Methods*; *SI Appendix*, section S4). Dividing S by the thickness of sediment deposits, which necessarily exceeds the saturated aquifer thickness, provides lower-bound estimates of specific storage spanning $10^{-6}$ to $10^{-4}$ m^−1^ with a logarithmic mean of $7\times 10^{-6}$ m^−1^. These values agree with previous point measurements in the Central Valley (29) and with the typical ranges of $10^{-5}$ to $10^{-4}$ m^−1^ for unconsolidated materials like sand, silt, and clay and $10^{-6}$ to $10^{-5}$ m^−1^ for consolidated materials like sedimentary rocks (61), which is consistent with the heterogeneous, sedimentary nature of the Sacramento Valley.

Altogether, these spatially heterogeneous storage characteristics indicate that at most 18 to 42 km^3^ of groundwater is elastically stored within the area sampled by our data and that 0.2 to 0.5 km^3^/y of this volume was extracted and recharged annually from 2016 to 2021 (Estimating groundwater volumes in *Materials and Methods*). These values represent only the elastic component of the total storage capacity and exclude the much larger contribution from specific yield, which dominates storage in the shallow unconfined aquifer layers (30). This annual release from elastic storage is slightly larger than the 1963–2003 average of ∼0.1 km^3^ estimated through hydrologic modeling (29). The residual subsidence rates observed in 2021–2022 (Fig. 4*F*), here assumed to be entirely inelastic, amount to a permanent storage loss of 0.2 km^3^/y, representing a five-fold increase from the 2016–2021 rate of 0.04 km^3^/y. This annual loss is equivalent to 4% of Shasta Lake, California’s largest water reservoir, or 30% of Los Angeles’ annual water consumption. Regional-scale estimates derived from GRACE/-FO (9) and joint GNSS-GRACE/-FO inversions (62) suggest that this accelerated groundwater loss extends beyond the Sacramento Valley to the entire Central Valley.

## Conclusions and Perspectives

Characterizing surface deformation in the Sacramento Valley reveals that a significant fraction of the aquifer system abruptly transitioned from a primarily poroelastic regime during the 2016–2020 interdrought period to a primarily inelastic, aquifer-damaging regime in 2021, in all evidence due to increased groundwater extraction during the 2020–2022 drought. The resulting loss of storage capacity imaged here, along with the likely reduction in permeability and well yield, may warrant the critically overdrafted designation and underscores the urgent need for more conservative groundwater management practices. While this compaction will never recover, the recent surge in precipitation in California brings hope that the aquifer system has or will soon transition back to a primarily poroelastic regime in response to decreased groundwater demand and increased recharge. Nonetheless, we anticipate the thicker and lower-permeability sedimentary units to undergo delayed compaction for years to come, implying that overexploitation of groundwater during the 2020–2022 drought may have long-lasting consequences for land subsidence in the Sacramento Valley.

More broadly, our analysis demonstrates that, because of the highly heterogeneous nature of aquifer systems, even a relatively dense groundwater monitoring network was insufficient to anticipate and diagnose the onset of widespread inelastic compaction. Real-time monitoring of surface displacements through satellite geodesy, which provide an integrated measure of internal deformation over the entire sedimentary column, could have enabled early detection of this sharp transition and potentially reduced storage loss through timely remedial actions. The development of multitechnique hydrogeophysical observatories with colocated, depth-sensitive geophysical and hydrological instruments would help map the internal structure of aquifer systems, constrain physical hydromechanical models and, hence, further our mechanistic understanding of this critical transition, help calibrate satellite observations, and enable more accurate space-based monitoring of groundwater resources worldwide. Developing such capability is especially critical in regions where in situ monitoring is unviable but groundwater resources increasingly vital (2).

## Materials and Methods

### Lithology.

We use the Central Valley 3D texture model from ref. 30 (https://www.sciencebase.gov/catalog/item/61fc85a2d34e622189cc090e), updated from ref. 29, to characterize the distribution of fine-grained materials in the Sacramento Valley. The texture model was obtained by interpolating thousands of lithologic logs through kriging. We average the percentage of fine-grained materials in the upper 400 m of the aquifer system, as most extraction wells and lithologic logs do not extend deeper than 400 m in the Sacramento Valley.

### InSAR.

We build Line-Of-Sight (LOS) deformation time series from the descending (track 115) Synthetic Aperture Radar images acquired by the Sentinel 1A-B satellites between January 2016 and September 2022 with a small-baseline subset (SBAS) approach. All interferograms between an acquisition and its 6 consecutive acquisitions are built (*SI Appendix*, Fig. S1.1) to reduce potential unwrapping error propagation while keeping a tractable number of interferograms (63). We coregister images and build interferograms using the ISCE package (64, 65). To improve interferometric coherence, we apply spatial averaging (i.e. multilooking) leading to a final pixel size of ∼74 m by 112 m, tropospheric corrections using ECMWF-ERA 5 global reanalysis (66), and reasonable spatial filtering (67) before unwrapping with a branch-cut algorithm (68) areas with a coherence above 0.4. Potential unwrapping errors are corrected (69) and the best-fitting plane is subtracted from each interferogram to remove long-wavelength signals originating from residual atmosphere or orbital errors.

The temporal evolution of phase change, hence deformation in LOS, is computed from unwrapped interferograms with a Kalman Filter time series analysis (KFTS) (42). KFTS solves together for the phase change $\phi_{k}$, at each time step $t_{k}$ with respect to the first date, with its uncertainty and for the parameters $a_{i}$ of a functional model of deformation, which is used to forecast deformation, whenever and wherever interferograms could not unwrap. KFTS is a tractable method in time by nature (data assimilation) and is well suited for future systematic monitoring of aquifers. We chose a model including a linear trend and seasonal oscillations, which writes as

[1] $$\begin{array}{c} \phi_{k}=a_{0}+a_{1}t_{k}+a_{2}\sin\left(\omega t_{k}\right)+a_{3}\cos\left(\omega t_{k}\right)+\gamma , \end{array}$$

where ω is the annual frequency and γ the mismodeling error accounting for temporally uncorrelated signal arising from residual atmospheric noise. We set its SD $\sigma_{\gamma }$ to 2 cm. To initialize KFTS, we set an a priori null model with SDs of 4 cm on $a_{0}$, 3 cm/y on $a_{1}$ and 1 cm on $a_{2,3}$, following the guidelines of ref. 42. This a priori helps regularize the problem and avoid overfitting when insufficient data are available.

To estimate vertical deformation, we project the resulting descending time series (i.e. multiply LOS by the inverse of the incidence angle cosine) ignoring ascending data, as they are subject to greater atmospheric noise associated with the local time of acquisition corresponding to late afternoon (*SI Appendix*, Fig. S1.3). The estimated displacements are relative to a stable area with high coherence (black star in Fig. 1*B*). Note that the resulting InSAR time series are fully independent of GNSS. See *SI Appendix*, section S1 for more details on the processing, accuracy, and precision of the InSAR dataset.

### GNSS.

We use vertical daily GNSS station position time series processed by the Nevada Geodetic Laboratory (NGL) at the University of Nevada (70). NGL uses the GipsyX 1.0 software (71) from the Jet Propulsion Laboratory (JPL) to compute position time series based on single-station precise point positioning (PPP) with carrier phase ambiguity resolution (72). Satellite orbit and clock used for the PPP processing are JPL’s Repro 3.0 products. All products are based on, and solutions are aligned to, the IGS14 realization (73) of the ITRF14 reference frame (74). The VMF1 mapping function with elevation-dependent data weight, and with nominal dry and wet zenith delays from the numerical weather model European Centre for Medium-Range Weather Forecasts (75) is used to correct tropospheric effects. For more details on NGL’s processing strategy, see https://geodesy.unr.edu/.

GNSS station position time series are then postprocessed by JPL as described in ref. 76. Elastic displacement produced by nontidal atmospheric and nontidal oceanic loading is removed from the GPS series using predictions from GFZ (77). Nontidal atmospheric loading produces displacements of several mm each day. Removing this deformation reduces the root mean square vertical misfit by about 12% in California (78). Offsets in the evolution of position caused by logged and suspected antenna substitutions are identified and removed by fitting them with a linear and a 1-year sine term, as well as potential offsets following the method of ref. 79. Note that because active tectonic vertical motions are likely small in the Central valley (80), and difficult to estimate precisely, we do not correct our geodetic observations for any tectonic contributions.

We select the 25 stations within the boundaries of the Sacramento Valley with at least 2.5 y of data from Jan. 2016 to Oct. 2022.

#### Groundwater levels.

We use the monthly averaged continuous time series provided by the DWR (https://data.cnra.ca.gov/dataset/continuous-groundwater-level-measurements) and take the monthly average of the provided periodic measurements (https://data.cnra.ca.gov/dataset/periodic-groundwater-level-measurements). Of the 435 continuous and 3,966 periodic wells located in the study area, we retain the 2,531 wells with at least 3 data points during the study period of Oct. 2015 to Oct. 2022 for the modern groundwater analysis (Figs. 2 and 3). We also retain the 241 wells with 1) a first observation before 1980, 2) a last observation after 2021.5, 3) at least 50 observations, and 4) a known-well depth deeper than 25 m (such that the well is likely in the confined or semiconfined part of the aquifer system), for the historic groundwater analysis (Figs. 4 and 5).

### GRACE/-FO.

We use monthly global measurements of the space and time varying Earth’s gravity field recorded by the Gravity Recovery And Climate Experiment (GRACE), from April 2002 to June 2017 (10), and in-orbit GRACE-Follow On (GRACE-FO), from June 2018 to October 2022 (11). In particular, we use the solution of ref. 43—based on previous work by ref. 81. This solution uses Multichannel Singular Spectrum Analysis (M-SSA) (82) to exploit spatiotemporal information of multiple Level-2 GRACE/-FO solutions, expressed in Stokes coefficients of the gravity fields spherical harmonics decomposition, filtered using the DDK7 decorrelation and complementary Lobe-Edge filter (43, 83, 84), to fill observational gaps and filter North–South striping artifacts caused by instrumental and processing errors. More precisely, missing data in GRACE/-FO Equivalent Water Height (EWH) time series processed by the Center of Space Research (CSR), GeoForschungsZentrum (GFZ), Institute of Geodesy of the University of Graz (GRAZ), and Jet Propulsion Laboratory (JPL), and outliers are replaced using M-SSA iteratively to obtain a combined evenly sampled solution (85). Then, M-SSA is applied to retrieve common signals between each EWH time series and its same-latitude neighbors to further reduce residual spatially uncorrelated noise. Note that the GRACE/-FO M-SSA solution is corrected for nontidal high-frequency atmospheric and oceanic mass variation models, namely the Atmosphere and Ocean Dealiasing Level-1B (AOD1B) model (86), which is consistent with our GNSS postprocessing strategy.

### GRACE/-FO Deformation Model.

We compute Earth’s surface elastic deformation resulting from the hydrological loading inferred from GRACE/-FO M-SSA (43) solution at the GNSS sites and over the InSAR track following the method proposed by ref. 48. We decompose the GRACE/-FO-derived surface loads into spherical harmonics coefficients and use the Love numbers theory (87) to predict surface displacements induced by surface loading on a purely elastic Earth structure based on the Preliminary Reference Earth Model (PREM) (88). The degree-1 coefficients of the Earth’s gravity field—which are not measured by the GRACE satellites—are inverted from residual time series of a global network of GNSS stations and the GRACE derived loading model from 2003 to 2017 (48), and then estimated over the 2006–2022 period by fitting a 1-y sinusoid to the degree-1 deformation field.

### Groundwater Projections.

To solve for the best-fit scaling factor between an individual groundwater or geodetic time series, X, and the mean groundwater time series, $\overset{¯}{X}$, we project X onto $\overset{¯}{X}$ using standard vector projection:

[2] $$\begin{array}{c} X_{\mathrm{proj}}=\left(\frac{X\cdot \overset{¯}{X}}{{\left\|\overset{¯}{X}\right\|}^{2}}\right)\overset{¯}{X}. \end{array}$$

Here, $X_{\mathrm{proj}}$ is the resulting projected time series, and the scalar quantity $\frac{X\cdot \overset{¯}{X}}{{\left\|\overset{¯}{X}\right\|}^{2}}$ is the projection amplitude, which we use to construct the spatial distributions in Fig. 3 *A* and *C*. We interpolate $\overset{¯}{X}$ to the epochs of X such that X and $\overset{¯}{X}$ have the same temporal sampling and dimensions.

To obtain reliable amplitudes for the groundwater dataset, we only perform projections on the 2,196 wells with at least 10 data points spanning at least 2.5 y and retain the 2,145 wells with a positive projection amplitude. We interpolate the 2,145 point measurements to estimate groundwater variations over the entire Sacramento Valley with a Delaunay triangulation-based natural neighbor algorithm (89). The spatial interpolation should be quite reliable given the high density of observations in most areas of the valley.

### Elastic Storage Capacity.

We estimate the uniaxial storage capacity S of the aquifer from the ratio of vertical displacement $u_{z}$ to change in groundwater level Δh as

[3] $$\begin{array}{c} S=\frac{3(1-\nu_{u})}{B(1+\nu_{u})}\frac{u_{z}}{\Delta h}, \end{array}$$

where $\nu_{u}$ is the undrained Poisson’s ratio and B is Skempton’s coefficient (4). See *SI Appendix*, section S4 for a complete derivation of Eq. **3**. A reasonable range of values for B and $\nu_{u}$ for a sedimentary aquifer basin is 0.7 to 1.0 and 0.3 to 0.5, respectively (4). Using $B=1.0,\nu_{u}=0.5$ and $B=0.7,\nu_{u}=0.3$ in Eq. **3** yields an expected range of values for S of:

[4] $$\begin{array}{c} S=\frac{u_{z}}{\Delta h}\;\text{to}\;2.31\frac{u_{z}}{\Delta h} \end{array}$$

The lower bound, $S=u_{z}/\Delta h$, corresponds to the incompressible-constituents limit often assumed in hydrogeology and soil mechanics (90). In this limit, the pore water and solid grains are assumed to have negligible compressibility compared to that of the porous matrix.

### Estimating Groundwater Volumes.

The volume of water released from elastic storage can be obtained as

[5] $$\begin{array}{c} \Delta V_{w,el}=\frac{SV\Delta h}{b}=SA\Delta h, \end{array}$$

where V, b, and A are the aquifer volume, thickness, and area, respectively. We note that $\Delta V_{w,el}$ is simply equal to $u_{z}A$ when S is taken as $u_{z}/\Delta h$. To estimate the mean annual $\Delta V_{w,el}$ released from elastic storage in the Sacramento Valley, we take the integral of SΔh over the area covered by the InSAR track for which we have an estimate of S, i.e.:

[6] $$\begin{array}{c} \Delta V_{w,el}=\sum_{i}^{n}S_{i}A_{\mathrm{pixel}}\Delta h_{i}, \end{array}$$

where n is the number of InSAR pixels, $A_{\mathrm{pixel}}$ is the area covered by one InSAR pixel (74 m × 112 m) and Δh is taken as the sum of seasonal groundwater variations (Fig. 3*A*) and groundwater trends (Fig. 3*B*). Using the range of S from $u_{z}/\Delta h$ to $2.31u_{z}/\Delta h$, we get a volume estimate of 0.20 to 0.47 km^3^.

Similarly, to estimate the volume of elastically stored water in the aquifer system, we use the formula:

[7] $$\begin{array}{c} V_{\mathrm{aquifer}}=\sum_{i}^{n}S_{i}A_{\mathrm{pixel}}b_{i}, \end{array}$$

where $b_{i}$ is the saturated aquifer thickness. Using the sediment thickness as a proxy for the maximum aquifer thickness (Fig. 1*E*) and the range $u_{z}/\Delta h$ to $2.31u_{z}/\Delta h$ for S, we obtain an upper bound estimate of $V_{\mathrm{aquifer}}=18$ to 42 km^3^.

Finally, to estimate the volume of water released from inelastic storage, $\Delta V_{w,in}$, we use:

[8] $$\begin{array}{c} \Delta V_{w,in}=\sum_{i}^{n}u_{z,in,i}A_{\mathrm{pixel}}, \end{array}$$

where $u_{z,in,i}$ is the inelastic subsidence at each InSAR pixel i. This yields $\Delta V_{w,in}$ estimates of 0.04 km^3^/y for 2016–2021 and 0.20 km^3^/y for Jan. 2021 to Oct. 2022.

## Supplementary Material

Appendix 01 (PDF)

## Data Availability

The processed GNSS, InSAR, and groundwater level datasets are available at https://doi.org/10.18715/IPGP.2023.ljsvz785 (91) and the GRACE/-FO M-SSA Level-3 solution (2003-01 to 2022-09) at https://doi.org/10.18715/IPGP.2023.lgquie56 (92).
