# Supplementary material for: Abrupt transition to irreversible damage in the overdrafted Sacramento Valley aquifer system
Source: Proc Natl Acad Sci U S A. 2026 Jul 27;123(31):e2526041123. doi: 10.1073/pnas.2526041123 (PMC13439106; doi:10.1073/pnas.2526041123)
Supplement: Supplementary file 1 [file pnas.2526041123.sapp.pdf]

PNAS

Supporting Information for

Abrupt transition to irreversible damage in the overdrafted Sacramento Valley aquifer system

Stacy Larochelle, Kristel Chanard, Manon Dalaison, Jérôme Fortin, Romain Jolivet, Laurent Longuevergne, Luce Fleitout, Donald F. Argus, Louis-Marie Gauer, and Jean-Philippe Avouac.

Jean-Philippe Avouac, Stacy Larochelle
E-mail: stacy.larochelle@epss.ucla.edu, avouac@caltech.edu

This PDF file includes:

Supporting text S1 to S4
Fig. S1.1 to S4.1
SI References

Supporting Information Text

S1. Geodetic datasets

InSAR accuracy and precision. The rigorous propagation of uncertainties through the interferometric network and model forecast is key in estimating and assessing the reliability of deformation in our regions of interest where data is often discontinuous. Fig. S1.2 illustrates different metrics reflecting the accuracy and precision of the descending InSAR measurements in and around the Sacramento Valley.

The root mean square (RMS) misfit between data (interferograms) and reconstituted phase difference (Fig. S1.2B) quantifies the agreement between the estimated time series and the available interferograms, which may contain inconsistencies. These inconsistencies are non-zero closure phase due to, mostly, deficient unwrapping (1), but also some multilooking biases from soil moisture variations or vegetation changes (2, 3). In most regions it is less than 1 cm on average.

The estimated standard deviation (STD) on time series of phase changes (Fig. S1.2C) is mostly a result of the interferometric network density and connectivity (see similarity with S1.2A). Indeed, on a given pixel, located in an agricultural field, many interferograms may be missing and this will directly impact the posterior standard deviation of phase change (median in Fig. S1.2C, error bars in time series of Fig. 3D) Fig. S1.3 shows similar metrics for the ascending InSAR measurements, which we do not use in this study. Indeed, we processed the ascending track 35 together with the descending track 115 of Sentinel-1 with the same parameters, however, our analysis revealed that the ascending track showed a large RMS misfit, the reason why we excluded this data from our analysis.

Using a simple bi-linear model in KFTS (4), including a rate change in 2021, we can theoretically propagate errors to the estimates of residual subsidence rate uncertainties (Fig. S1.2D,E). The slight negative correlation between the rate estimates seen in Fig. S1.2F results from the imposed continuity at 2021 – i.e., for the same time series, a larger inferred rate in 2016-2021 decreases the inferred rate in 2021-2022.

Nonetheless, we recognize that there may be some phase closure biases remaining in our time series (5). However, residual biases should not affect our results for several reasons. First, the studied signal is very large in amplitude (> 10 cm) and we do not interpret signal inferior to 2 cm. Second, our error estimation accounts for missing data due to coherence loss affecting vegetated grounds. Then, the agreement with GNSS within 2 cm is a validation that we can trust our InSAR to that level. Lastly, in the hypothesis of strong biases affecting pixels away from any GNSS station, we would expect this bias to mostly appear as an apparent constant velocity throughout the time series (2, 3). Thus, it cannot be mixed with the studied periodicity or the slope break in 2021 also observed at some GNSS stations.

InSAR comparison with GNSS and TRE Altamira processing. To evaluate the agreement between the InSAR and GNSS observations, we compute the RMSE between the two sets of LOS displacement time series. The resulting spatial distribution in Fig. S1.4A and the sample time series comparisons in Fig. S1.4B-G show that RMSE levels are similar in the Sacramento Valley and in the surrounding area (which should see negligible groundwater-induced deformation), except at two stations with anomalously high RMSE (P268 and P270; Fig. S1.4F,G). Discrepancies at these sites may arise from differences in signal spatial averaging inherent to each technique or from rapid changes in atmospheric or ground conditions, such as agricultural activities, which tend to affect InSAR more than GNSS (6), but are effectively addressed by our Kalman filter InSAR (4).

We also compare our InSAR solution to the TRE Altamira InSAR dataset commissioned by the California DWR (https://data.ca.gov/dataset/tre-altamira-insar-subsidence-data). While the TRE Altamira dataset has a more limited spatial coverage compared to our solution, we find that the two solutions generally agree well. Fig. S1.5 shows the excellent agreement between the two solutions at points near Artois (representative of Point 2 in Fig. 3D), Arbuckle (representative of Point 3 in Fig. 3D), and at GNSS station P271. Note that no TRE Altamira solution was available near Point 1.

GRACE/-FO deformation model. Fig. S1.6 illustrates how the GRACE/-FO modeled deformation compares to GNSS surface displacements. At station P344, located away from zones of intensive pumping, the observed GNSS displacements are in good agreement with the model, indicating that large-scale hydrological loading dominates the deformation field at those locations. In contrast, at station P267 located within a high-extraction zone, the model does not fit the observations well. This is because poroelastic deformation, which is not included in the GRACE/-FO model, dominates the deformation field at those locations.

Fig. S1.7 illustrates how the GRACE/-FO-modeled elastic loading deformation varies across the InSAR track. The deformation time series relative to the reference point – which is what is subtracted from the InSAR time series – shows that this is not a simple ramp signal, although they have relatively small amplitudes of 1-2 mm in this case.

S2. Groundwater levels

Modern groundwater levels. To characterize temporal fluctuations in water pressure in the aquifer system, we take the mean of detrended groundwater levels across all 2531 wells in the study area (Fig. S2.1A). We also consider how this mean groundwater time series varies as a function of depth in Fig. S2.1B-F by only using time series recorded at wells sampling a given depth interval. We find that this mean time series remains relatively constant below $\sim$25 m. The larger fluctuation amplitudes and consistent time series below $\sim$25 m suggest that the aquifer system, on average, transitions to relatively confined conditions around this depth. To characterize how these fluctuation amplitudes vary in space, we project each individual detrended groundwater time series onto the mean time series (Fig. 3A). Fig. S2.2 shows representative examples of these projections.

Historic groundwater levels. In Fig. 5, we consider all relatively complete historic time series to assess (1) whether or not they reached a new groundwater low between Jan. 2021 and Oct. 2022 and (2) whether they are associated with significant residual subsidence. Fig. S2.3 shows representative examples of these relatively complete historic time series. The four examples represent the four possible scenarios: (A) New groundwater low reached in 2021-2022 but no significant residual subsidence (0.6 cm/yr), (B) no new groundwater low reached in 2021-2022 and no significant residual subsidence (0.5 cm/yr), (C) new groundwater low reached in 2021-2022 and large residual subsidence (-11.6 cm/yr), (D) no new groundwater low reached in 2021-2022 but large residual subsidence (-10.8 cm/yr).

S3. Poroelastic and inelastic deformation

Poroelastic deformation. Fig. S3.1 shows examples of (detrended) InSAR time series projection onto the mean groundwater time series, highlighting that vertical deformation signals can be largely explained by the mean groundwater fluctuations observed at the regional scale. Fig. S3.4 and S3.6A show that the amplitude of the extracted GNSS and InSAR poroelastic deformation signals are in good agreement. The long-term poroelastic deformation estimated by multiplying the 2016-2022 groundwater trends (Fig. 3B) by the ratio of seasonal poroelastic deformation (Fig. 3C) to groundwater fluctuations (Fig. 3A) is shown in Fig. S3.2, revealing localized poroelastic subsidence rates of up to $\sim$2 cm/yr from 2016 to 2022.

Inelastic deformation. Fig. S3.3 shows examples of non-detrended InSAR time series vs inferred poroelastic (seasonal and long-term) deformation (similar to Fig. 3D), highlighting the large residual subsidence signals from Jan. 2016 to Oct. 2022. Fig. S3.5 and S3.6B,C show the generally good agreement between the residual subsidence rates estimated from the GNSS and InSAR time series, except at anomalous stations P268 and P270.

Fig. S3.7 compares the cumulative displacements between 2008 and 2017 measured by the campaign GPS survey (7) and the permanent GNSS stations in this study to the spatial distribution of accelerated residual subsidence measured by InSAR in 2021-2022.

Comparing the groundwater time series at the 96 wells located in zones of large residual subsidence rates ($\geq$ 15 cm/yr) to the associated InSAR time series confirms that groundwater levels recover during the winter and spring months, while the InSAR-measured displacements do not, indicative of inelastic deformation (Fig. S3.8). Similar to Fig. 4, Fig. S3.9 illustrates that the large residual subsidence rates observed in 2021-2022 do not necessarily correspond to new groundwater lows in the available records, although some of these well records may not be accurate as indicated by the station comments in the DWR database. We choose to still show these records as they are some of the few wells with historic records located in the zone of rapid subsidence.

S4. Elastic storage capacity

Here we derive expressions to estimate the uniaxial storage coefficient, S, in a poroelastic framework following Wang (2000) (8). We start from the constitutive equations for an isotropic poroelastic medium:

$$\sigma_{ij} = \frac{3K(1-2\nu)}{(1+\nu)}\epsilon_{ij} + \frac{3K\nu}{(1+\nu)}\epsilon_{kk}\delta_{ij} - \alpha p \delta_{ij}, \qquad [1]$$

which relates the stress components σ_{ij} to the strain components ϵ_{ij} and pore pressure p, given an elastic bulk moduli K, Poisson's ratio ν, and Biot-Willis coefficient α. Applying the mechanical conditions for an aquifer under uniaxial strain ($\epsilon_{xx} = \epsilon_{yy} = 0$) and constant vertical stress ($\sigma_{zz} = 0$) – as is usually assumed in hydrogeology – to the vertical component of Eq. 1 yields an expression for vertical strain ϵ_{zz} in terms of p:

$$\epsilon_{zz} = \frac{\alpha(1+\nu)}{3K(1-\nu)}p = \frac{\alpha}{K_v}p \qquad [2]$$

where $K_v = (1+\nu)/(3K(1-\nu))$ is the uniaxial bulk modulus. Defining pore pressure p in terms of groundwater level change, Δh, as $p = \rho g \Delta h$ and assuming constant vertical strain with depth such that $\epsilon_{zz} = u_z/b$, where u_z is the vertical displacement at the aquifer surface and b is the aquifer thickness, yields:

$$\frac{u_z}{\Delta h} = \frac{\alpha \rho g b}{K_v}. \qquad [3]$$

We note that when $\alpha = 1$, the term of the right-hand side of Eq. 3 is equivalent to the skeletal storage coefficient S_k often used in the land subsidence literature (9). For a poroelastic aquifer, the uniaxial specific storage S_s can be defined in terms of the bulk compressibility K_v, fluid compressibility K_f, and the "unjacketed" pore compressibility K_ϕ (8) as:

$$\frac{S_s}{\rho g} = \frac{\alpha^2}{K_v} + \phi\left(\frac{1}{K_f} - \frac{1}{K_\phi}\right), \qquad [4]$$

which reduces to the classic hydrogeologic definition of specific storage when $\alpha = 1$ and $1/K_\phi = 0$ (10):

$$\frac{S_s}{\rho g} = \frac{1}{K_v} + \frac{\phi}{K_f} \qquad [5]$$

S_s can also be expressed in terms of K_v and bulk poroelastic coefficients as:

$$\frac{S_s}{\rho g} = \frac{\alpha}{K_v}\frac{3(1-\nu_u)}{B(1+\nu_u)} \quad [6]$$

where B is Skempton's coefficient and ν_u is the undrained Poisson's ratio. Substituting Eq. 3 into 6 yields an expression for S_s in terms of measurable quantities u_z and Δh:

$$S_s = \frac{3(1-\nu_u)}{B(1+\nu_u)}\frac{u_z}{b\Delta h} \quad [7]$$

The uniaxial storage coefficient, S, related to the uniaxial specific storage, S_s, as $S = S_s b$ can then be obtained as:

$$S = \frac{3(1-\nu_u)}{B(1+\nu_u)}\frac{u_z}{\Delta h}. \quad [8]$$

Note that S is exactly equal to S_k in the incompressible-constituents limit in which $B = 1$ and $\nu_u = 0.5$. We also note that Wang (2000) reserves the symbol S to denote the poroelastic uniaxial specific storage and not the uniaxial storage coefficient as we do here.

Fig. S4.1 shows the lower-bound ($B = 1, \nu_u = 0.5$) and upper-bound ($B = 0.7, \nu_u = 0.3$) distributions of estimated storage coefficient S and specific storage S_s.

References

1. A Benoit, B Pinel-Puysségur, R Jolivet, C Lasserre, CorPhU: an algorithm based on phase closure for the correction of unwrapping errors InSAR interferometry. *Geophys. J. Int.* **221**, 1959–1970 (2020).
2. H Ansari, F De Zan, A Parizzi, Study of systematic bias in measuring surface deformation with SAR interferometry. *IEEE Transactions on Geosci. Remote. Sens.* **59**, 1285–1301 (2021).
3. Y Zheng, H Fattahi, P Agram, M Simons, P Rosen, On closure phase and systematic bias in multilooked SAR interferometry. *IEEE Transactions on Geosci. Remote. Sens.* (2022).
4. M Dalaison, R Jolivet, A Kalman filter time series analysis method for InSAR. *J. Geophys. Res. Solid Earth* **125**, e2019JB019150 (2020).
5. M Dalaison, B Pinel-Puysségur, R Jolivet, B Raimbault, L Drique, Relationship between closure phase error and interferometric coherence in multilooked InSAR in *AGU Fall Meeting Abstracts*, AGU Fall Meeting Abstracts. Vol. 2024, pp. G34A–08 (2024).
6. A Peltier, JL Froger, N Villeneuve, T Catry, Assessing the reliability and consistency of InSAR and GNSS data for retrieving 3D-displacement rapid changes, the example of the 2015 Piton de la Fournaise eruptions. *J. Volcanol. Geotherm. Res.* **344**, 106–120 (2017).
7. California Department of Water Resources, Technical Information Record (TIR) – NRO-2018-01: 2017 GPS Survey of the Sacramento Valley Subsidence Network (2018).
8. HF Wang, *Theory of Linear Poroelasticity with Applications to Geomechanics and Hydrogeology.* (Princeton University Press), p. 304 (2000).
9. R Smith, R Knight, Modeling land subsidence using InSAR and airborne electromagnetic data. *Water Resour. Res.* **55**, 2801–2819 (2019).
10. CW Fetter, *Applied Hydrogeology.* (Prentice Hall), 4th edition, (2001).

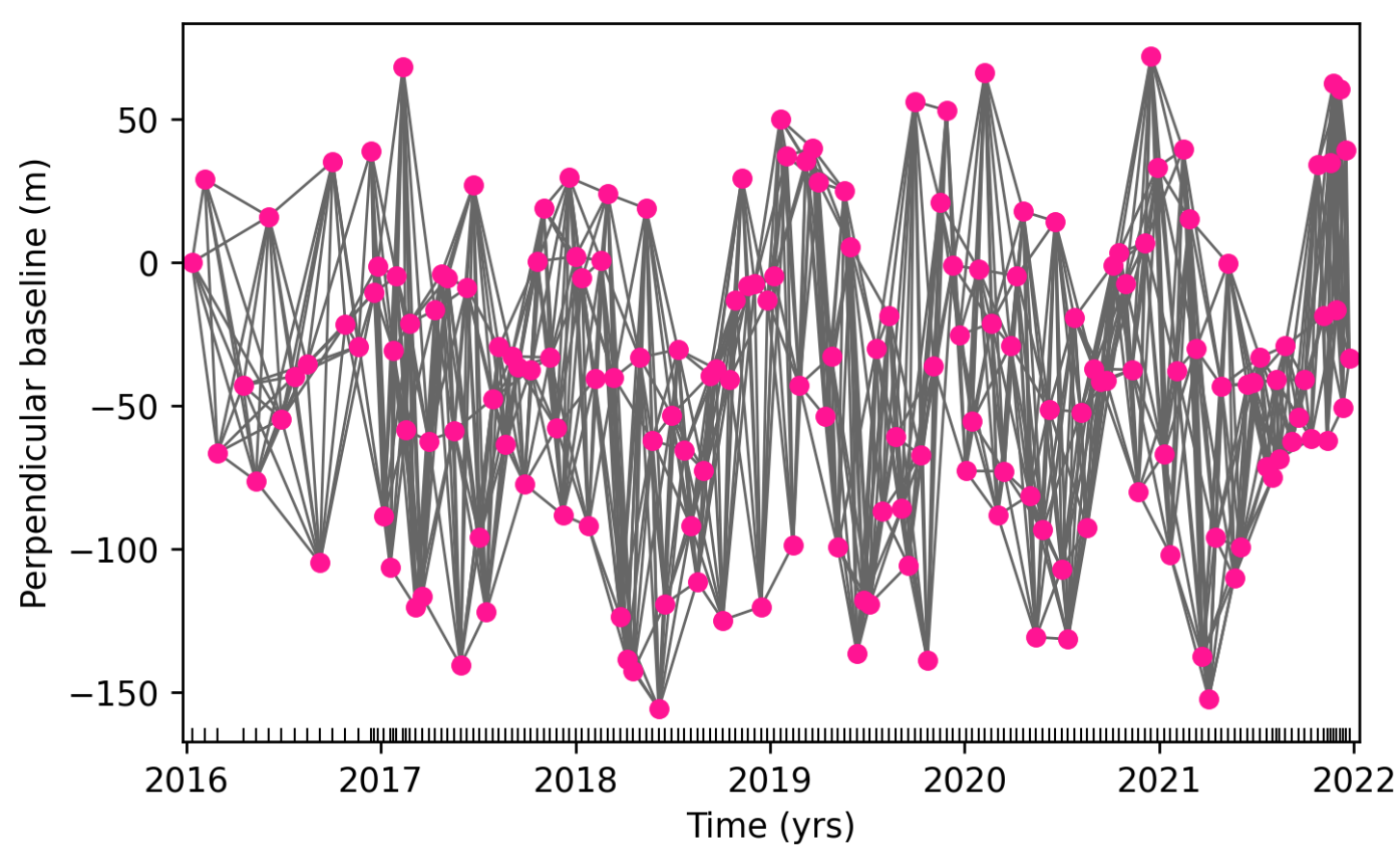

Fig. S1.1. InSAR interferometric network. Synthetic Aperture Radar images (pink dots) along descending track 115, are positioned as a function of the relative position of the satellite on its orbit and time during acquisition. Sentinel-1 acquisitions were on average regularly spaced every 12 days since 2017. Grey lines connect acquisitions involved in an interferogram. We constructed 1103 interferograms between 197 acquisitions, with temporal baseline ranging from 6 days to about 3 months.

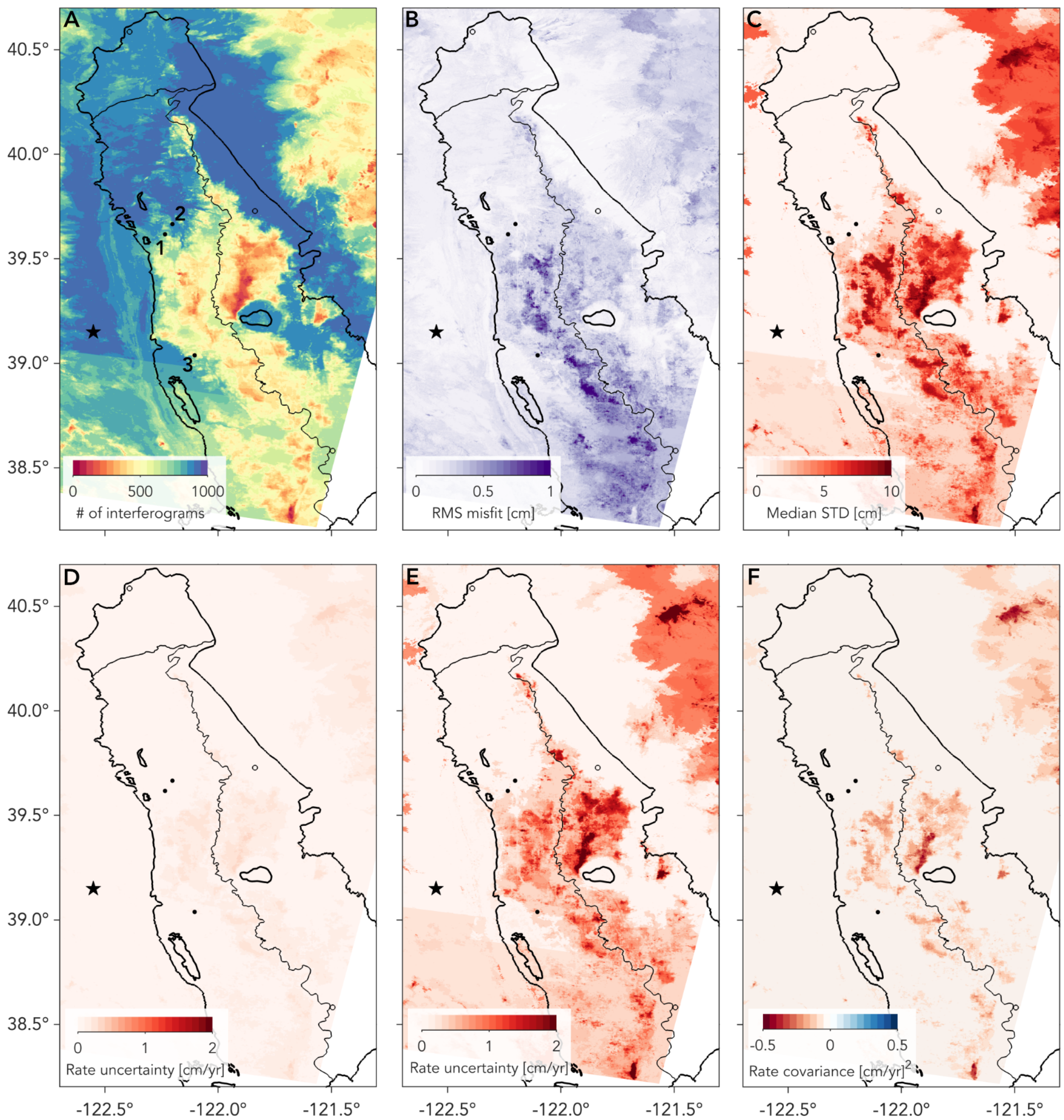

Fig. S1.2. Accuracy and precision of descending InSAR measures in and around the Sacramento valley. (A) Number of interferograms. (B) Root mean square misfit between data (interferograms) and reconstituted phase difference.(C) Median estimated standard deviation on time series of phase changes. (D,E) Standard deviation on residual subsidence rate between January 2016 and January 2021 (D) and January 2021 and October 2022 (E). (F) Covariance between the rate estimates. The black dots indicate the location of the three sample time series shown in Fig. 3D and the star indicates the reference point used for InSAR.

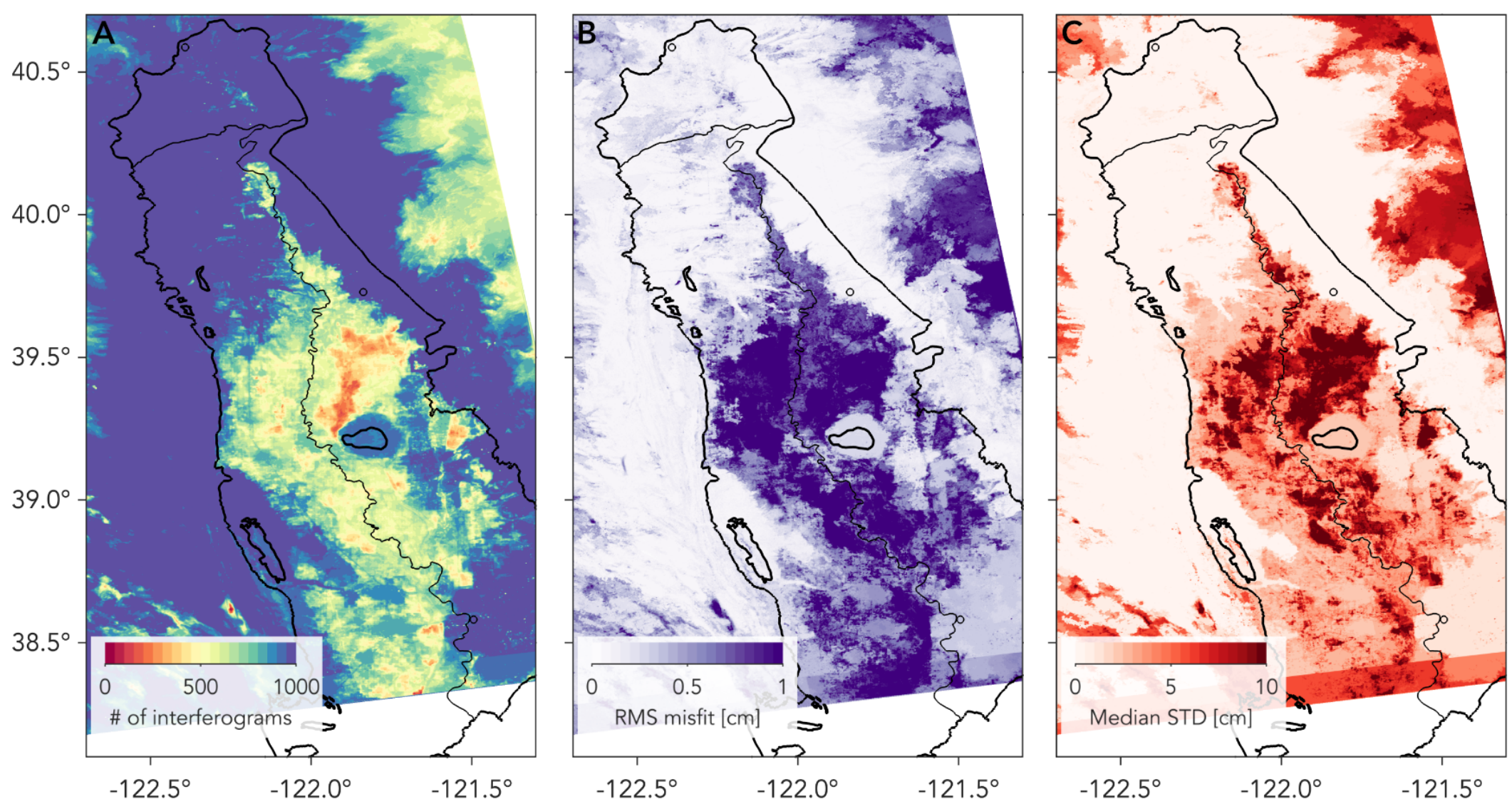

Fig. S1.3. Similar to Fig. S1.2A-C but for the ascending InSAR track.

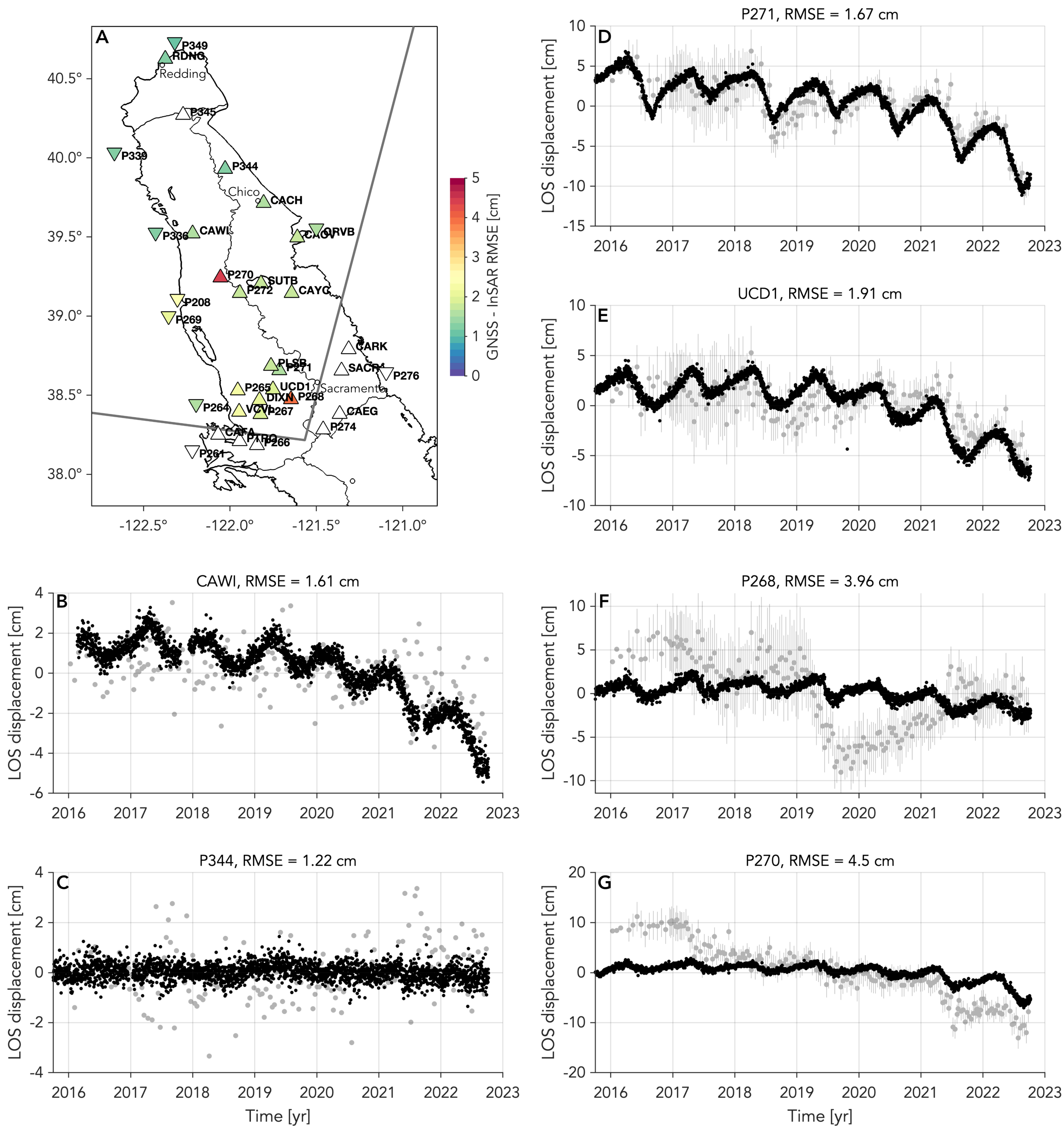

Fig. S1.4. (A) Root mean square error (RMSE) between LOS InSAR and GNSS displacement time series, both referenced to station P345, inside (upright triangles) and outside (downward triangles) the Sacramento Valley for GNSS stations located within the InSAR track. (B-G) Comparison of LOS GNSS and InSAR displacement time series at sample GNSS stations, including at the two stations with anomalous RMSE (P268 and P270).

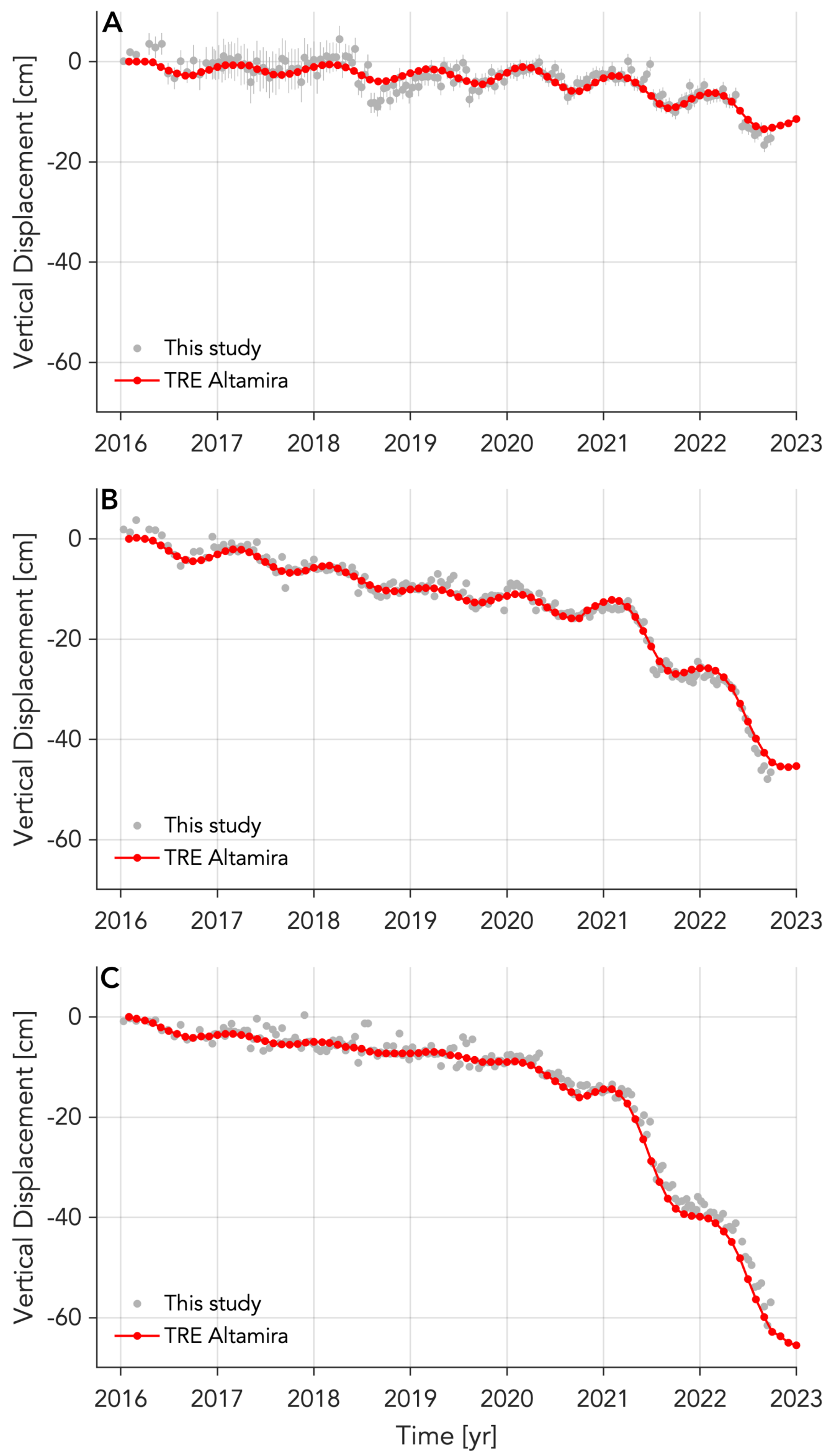

Fig. S1.5. Comparison of vertical InSAR displacement time series from this study (grey dots) and the TRE Altamira dataset (https://data.ca.gov/dataset/tre-altamira-insar-subsidence-data; red dots and curves) at (A) the location of GNSS station P271, (B) at a point near Artois, representative of Point 2 in Fig. 3D, and (C) at a point near Arbuckle, representative of Point 3 in Fig. 3D.

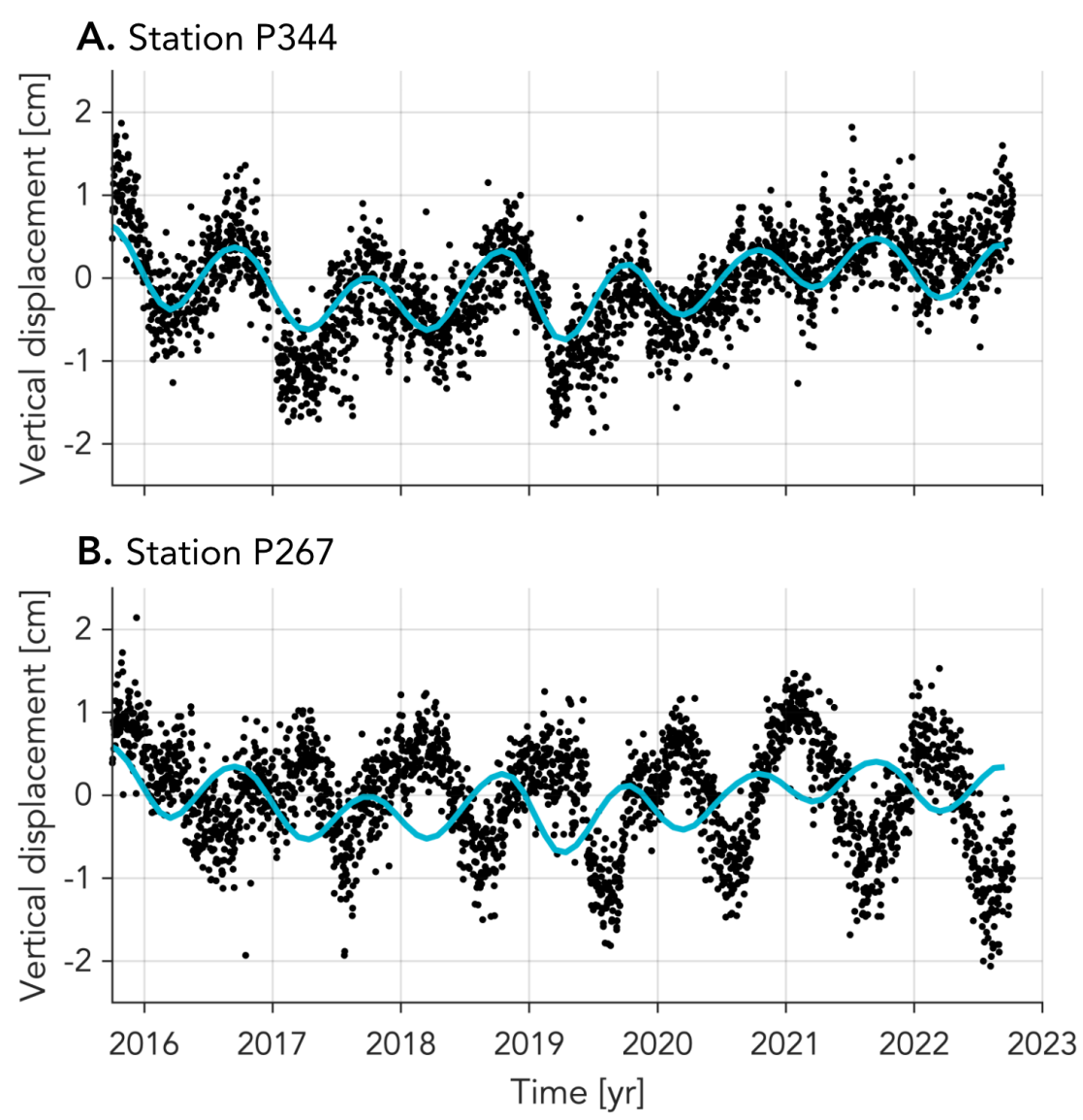

Fig. S1.6. Comparison of GNSS vertical displacements and GRACE model at GNSS stations P344 (A; dominated by elastic loading deformation) and P267 (B; dominated by poroelastic deformation).

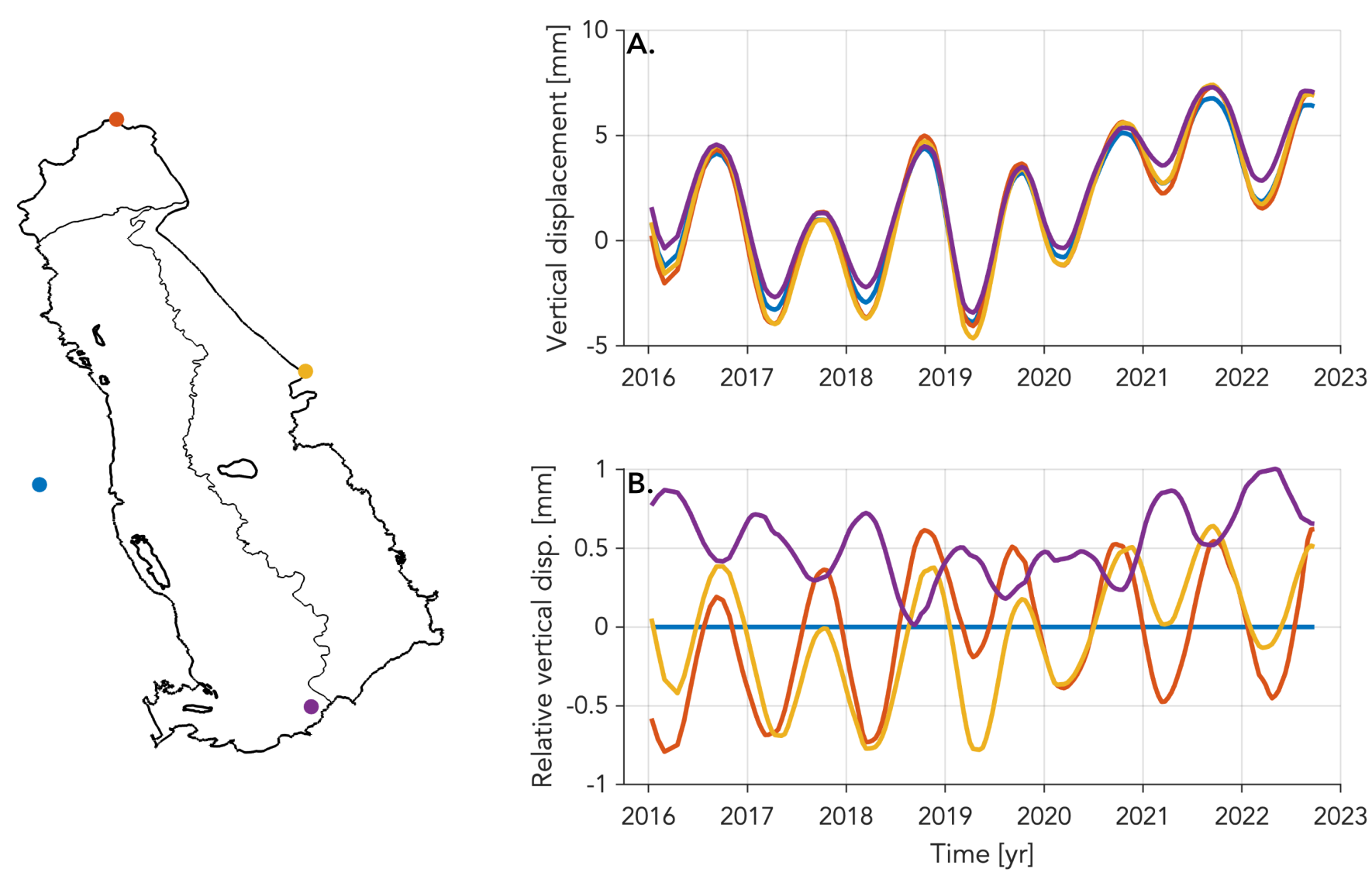

Fig. S1.7. Removing large-scale elastic loading deformation from the InSAR time series. (A) Vertical deformation from the GRACE/-FO elastic loading model at the four points shown on the map, including the InSAR reference point (blue). (B) Vertical deformation relative to the reference point. This is what is subtracted from the InSAR time series.

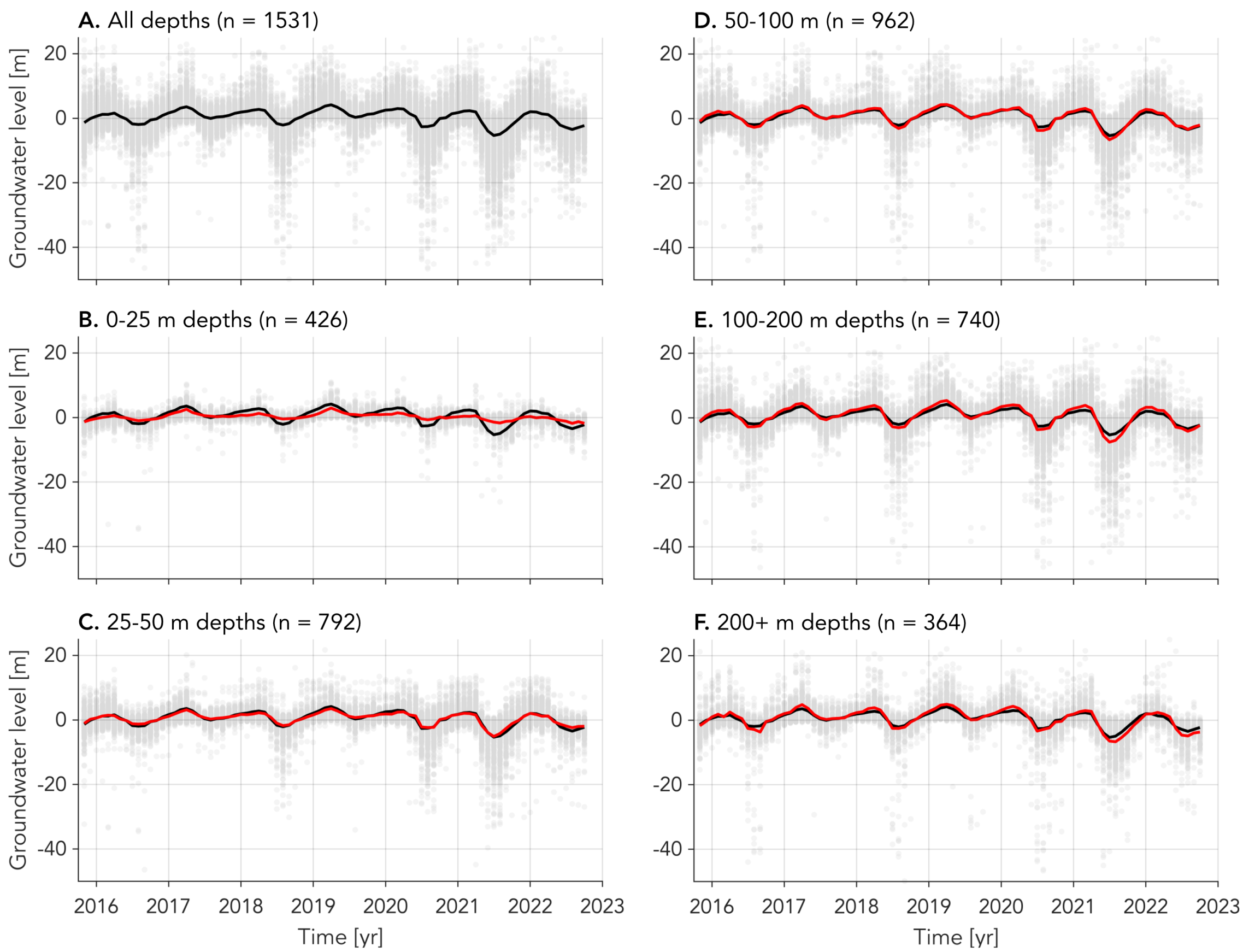

Fig. S2.1. (A) Detrended stacked (grey points) and mean groundwater level time series (black line) for all wells in the dataset. (B-F) Detrended stacked (grey points) and mean groundwater level time series for wells sampling a given depth interval (red line). The black line indicates the mean groundwater level time series for all wells in the dataset. Note that wells with specified screened intervals may appear in more than one depth bin.

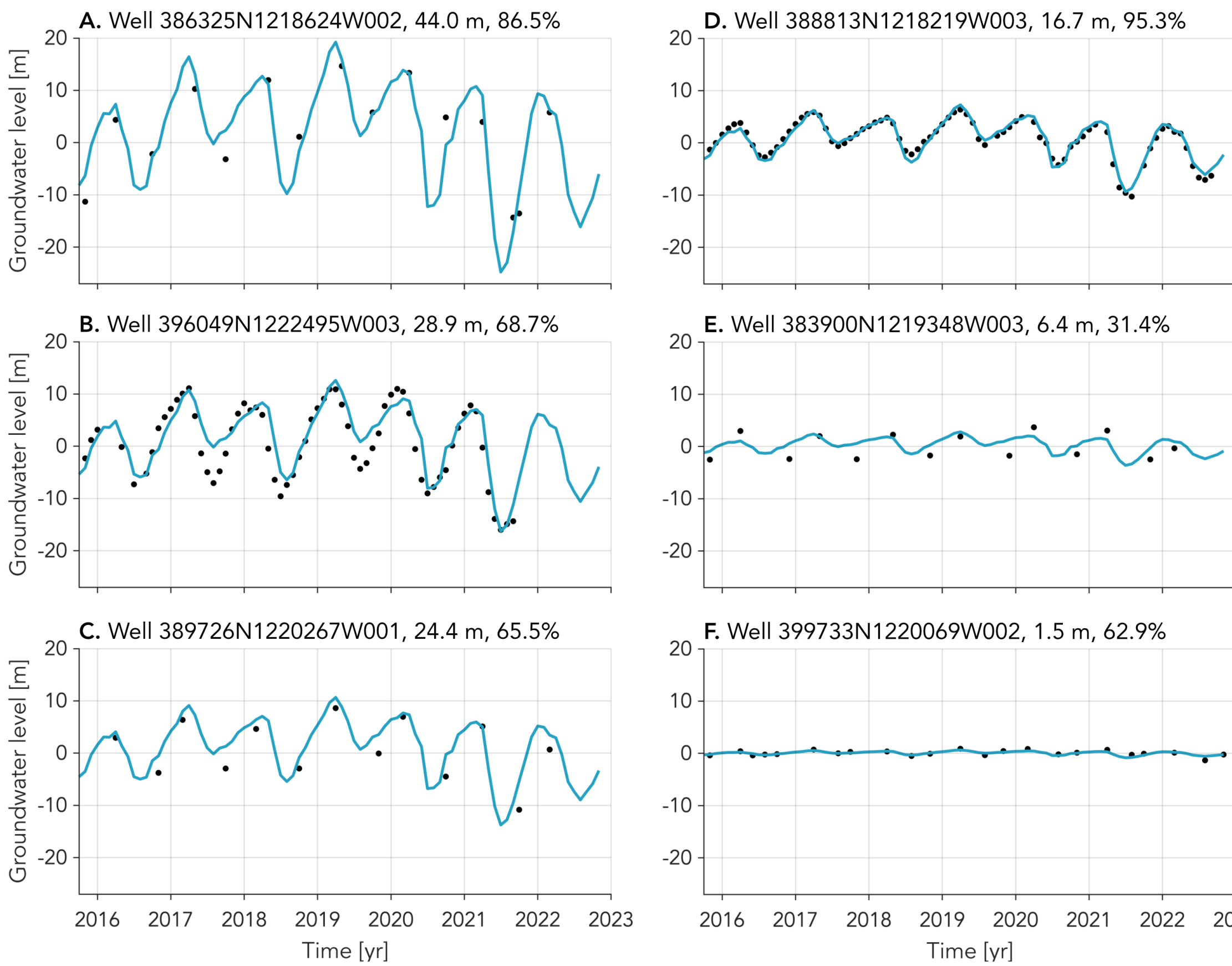

Fig. S2.2. Examples of projections of detrended groundwater level time series (black points) onto the mean groundwater time series (blue line).

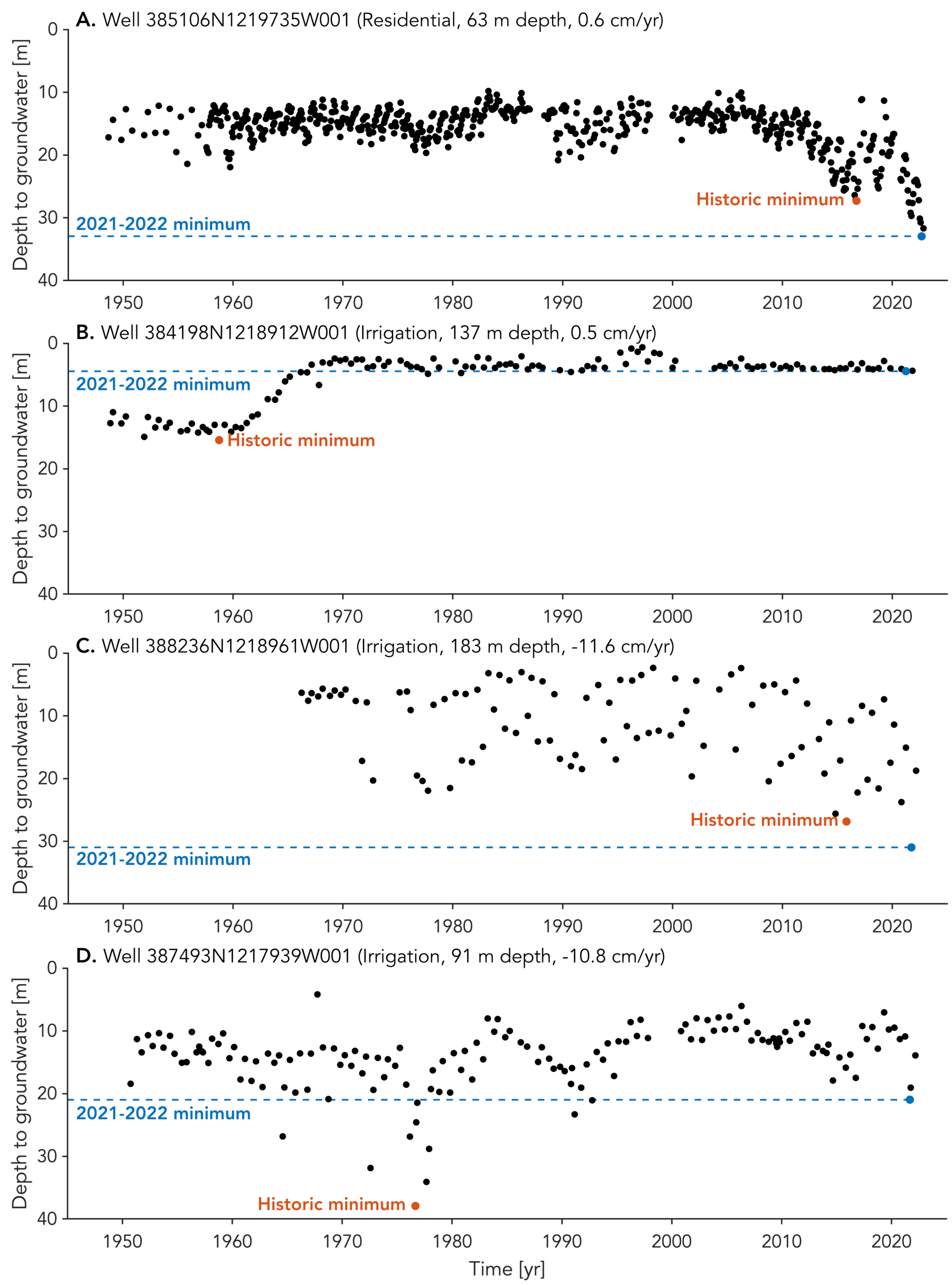

Fig. S2.3. Examples of historic groundwater level time series (black points). The historic groundwater low prior to 2021 is shown in orange and the groundwater low in 2021 and 2022 in blue.

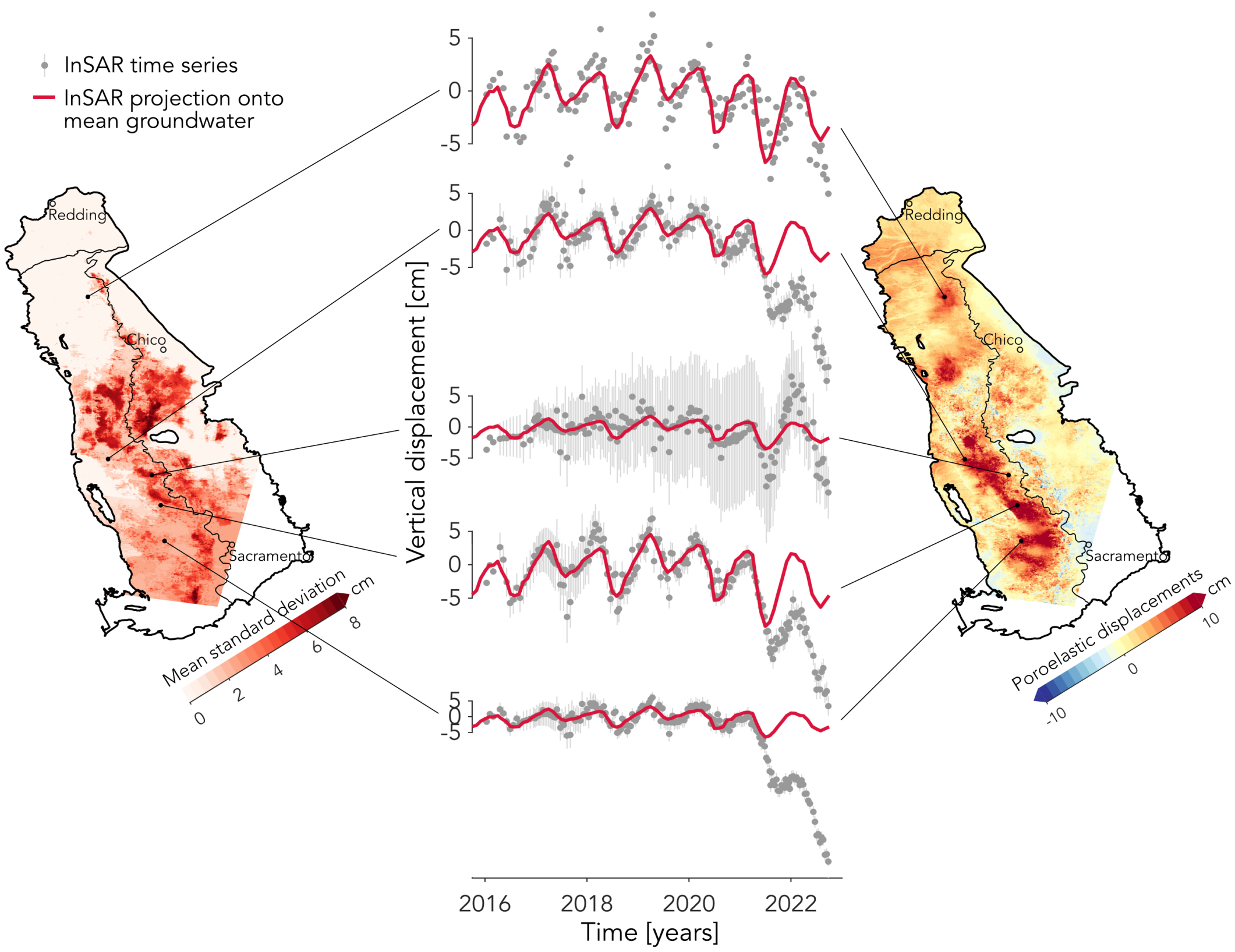

Fig. S3.1. Examples of InSAR projections onto mean groundwater time series and mean standard deviation map during 2016-2021. All time series have been detrended using the trend from 2016-2021.

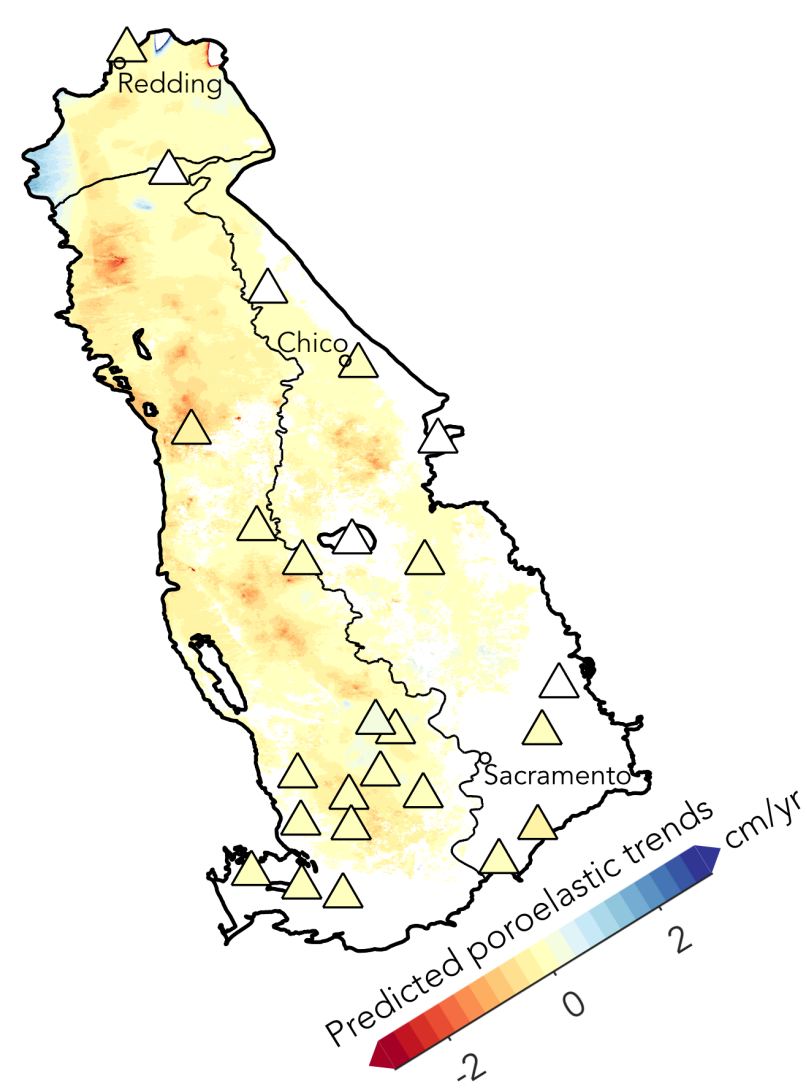

Fig. S3.2. Spatial distribution of predicted long-term poroelastic displacements.

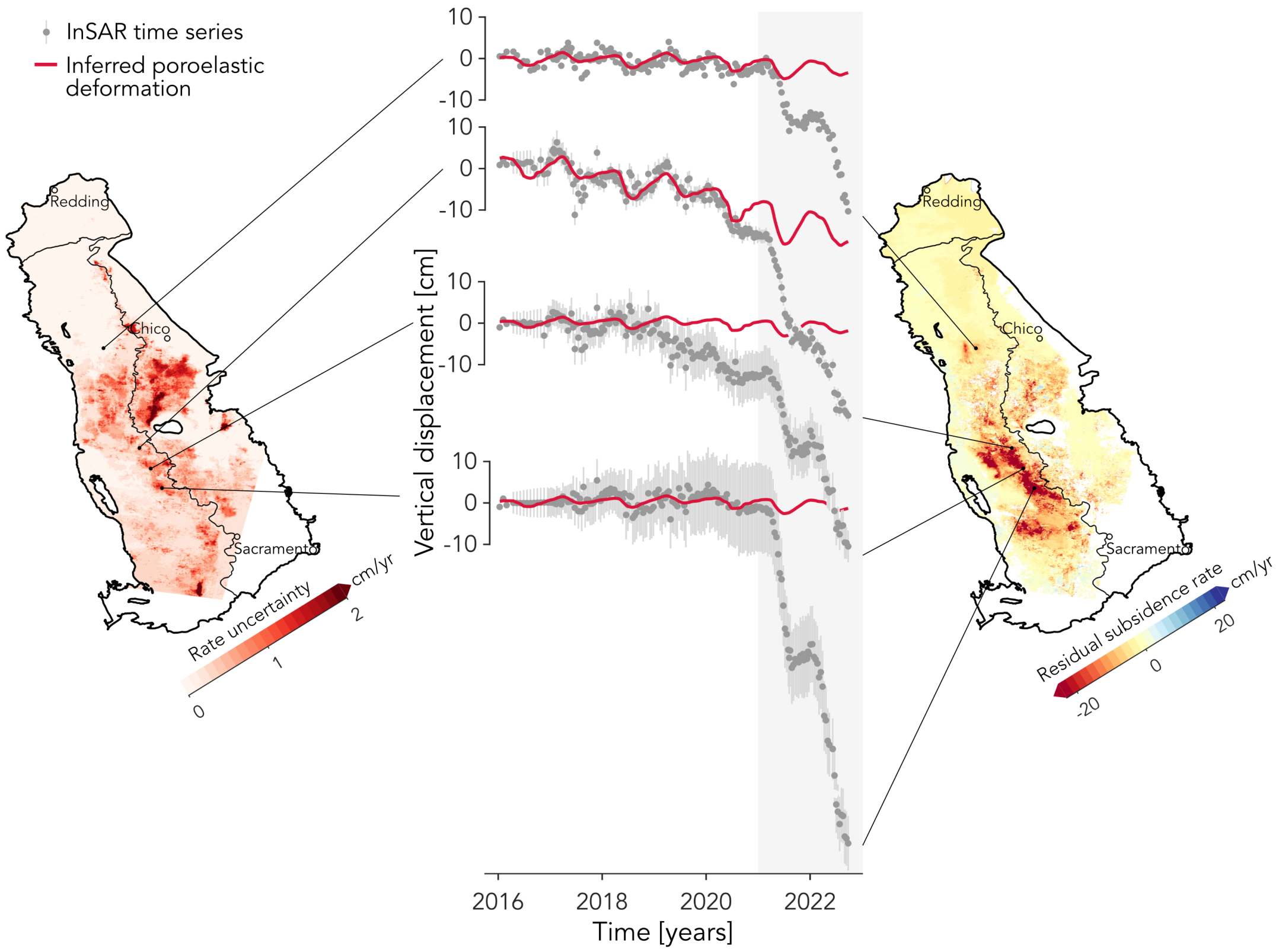

Fig. S3.3. Additional examples of InSAR vertical displacement time series vs inferred poroelastic deformation and rate uncertainty map for the 2021-2022 period, similar to Fig. 3D

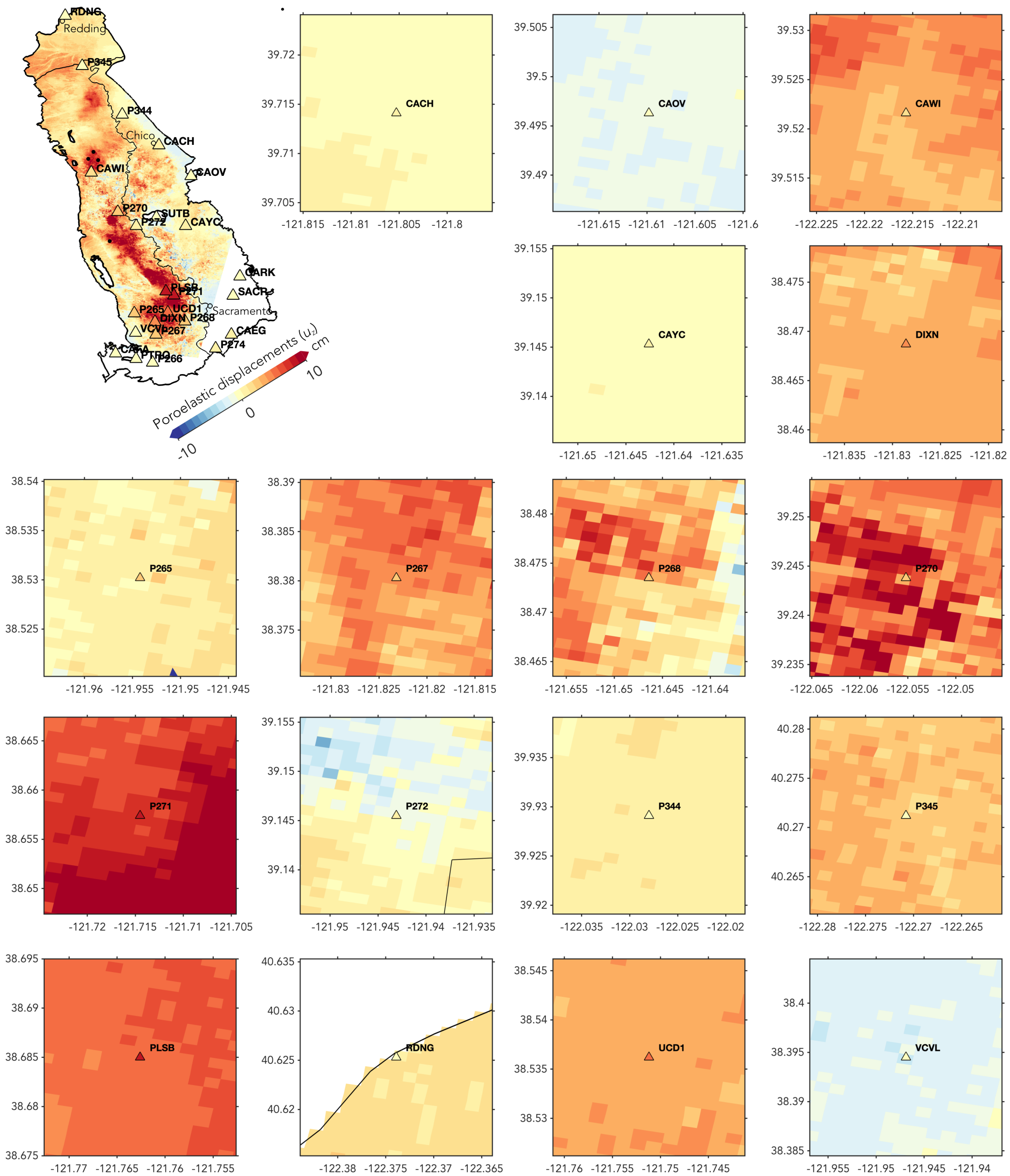

Fig. S3.4. Zoomed maps of Fig. 3A comparing GNSS and InSAR projection amplitudes at each GNSS station in the Sacramento Valley and within the InSAR track.

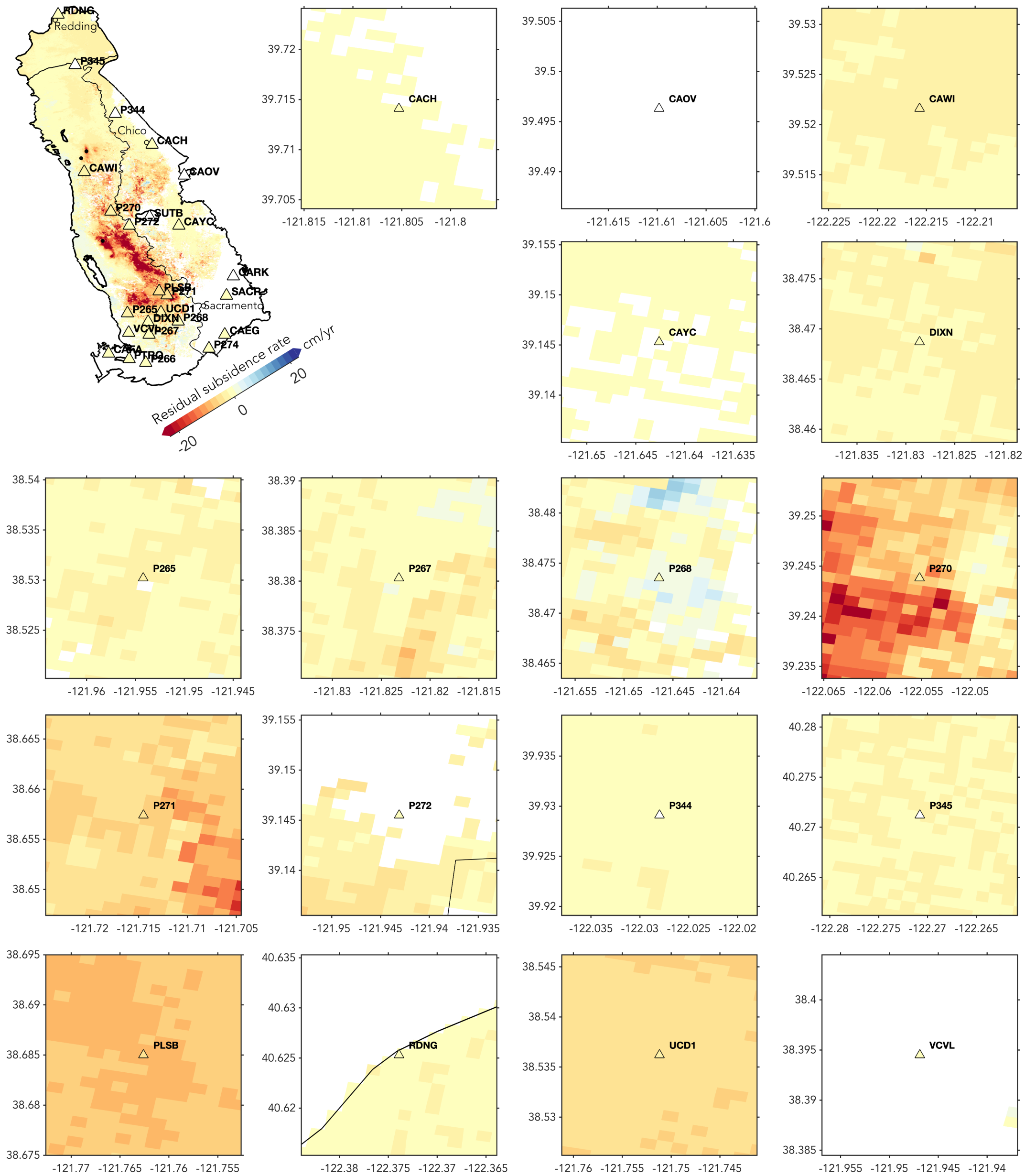

Fig. S3.5. Zoomed maps of Fig. 3E comparing GNSS and InSAR residual subsidence rates during 2021-2022 at each GNSS station in the Sacramento Valley and within the InSAR track. White InSAR pixels and GNSS stations indicate that no estimate of residual subsidence rate is available at this location.

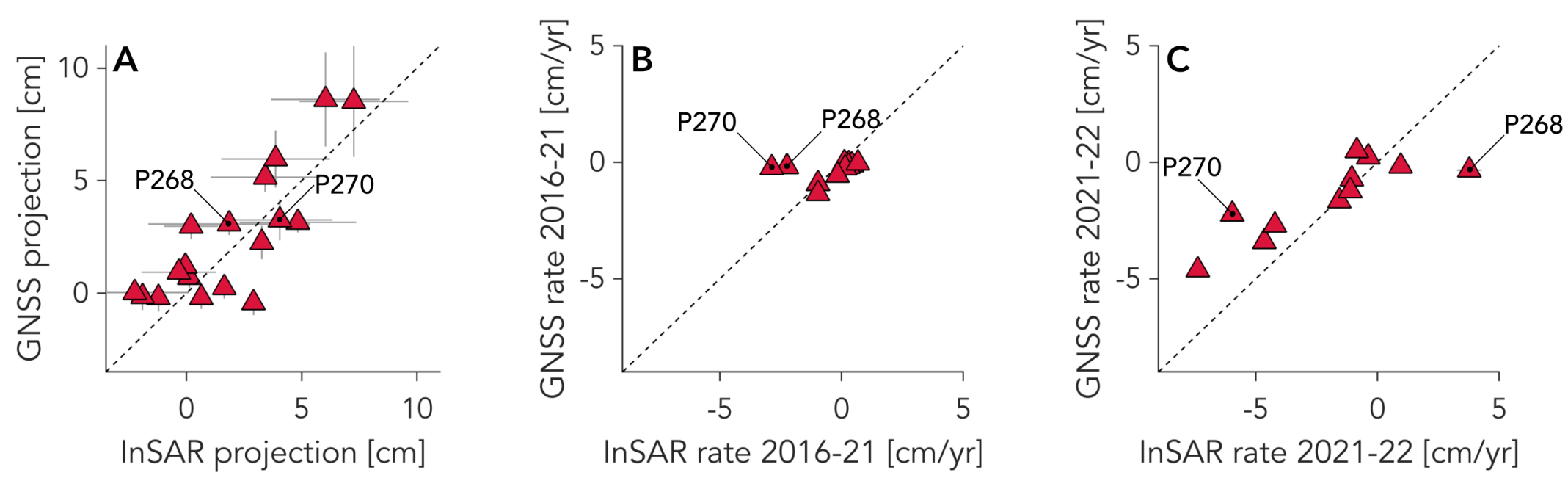

Fig. S3.6. Comparison of GNSS- and InSAR-inferred poroelastic amplitudes (A) and residual subsidence rates from (B) Jan. 2016 to Jan. 2021 and (C) Jan. 2021 to Oct. 2022. Stations P268 and P270 with anomalous RMSE (Fig. S1.4) are indicated in each plot.

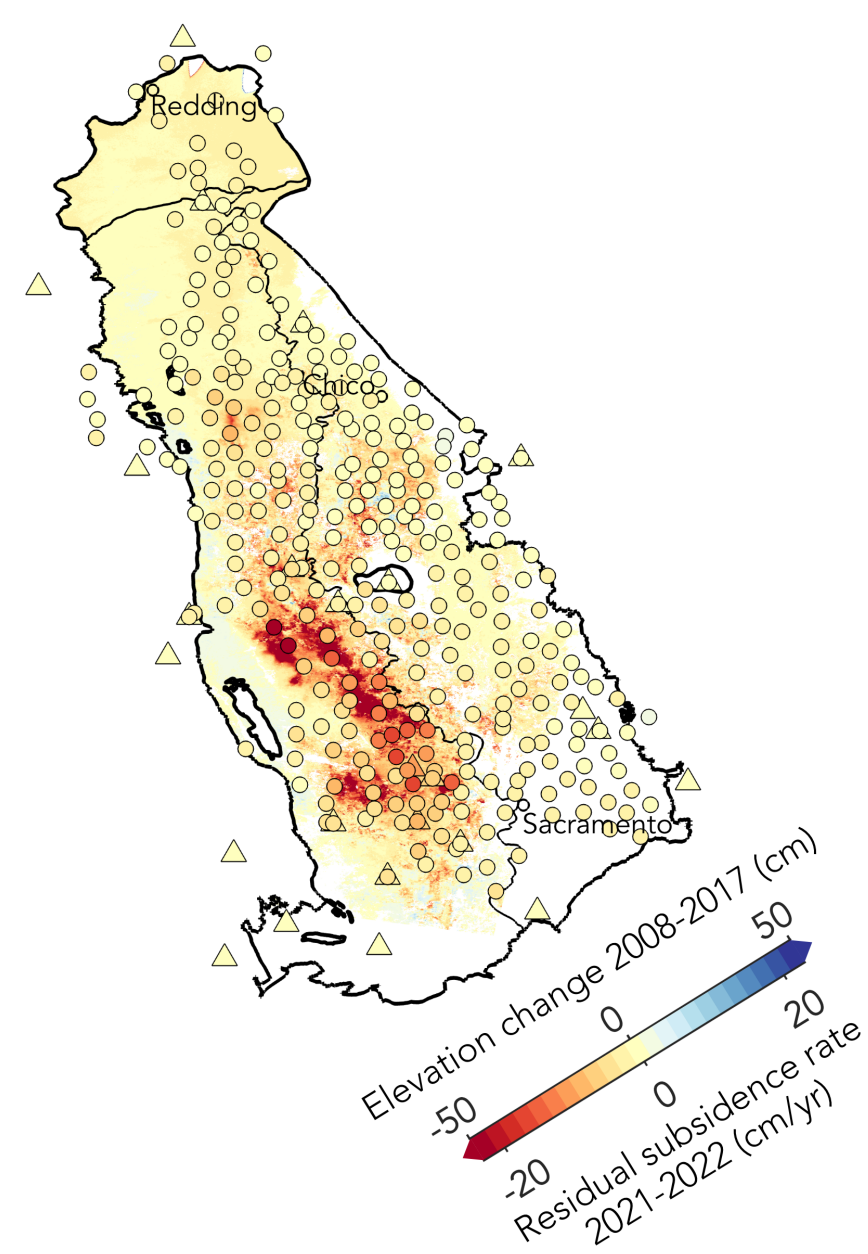

Fig. S3.7. Comparison with 2008-2017 campaign GPS subsidence survey. Total elevation change from 2008 to 2017 measured by the Sacramento Valley campaign GPS Subsidence Network (7) (circles) and by this study's permanent GPS stations (triangles) with the InSAR residual subsidence map from 2021-2022 (Fig. 3E) in the background for comparison purposes.

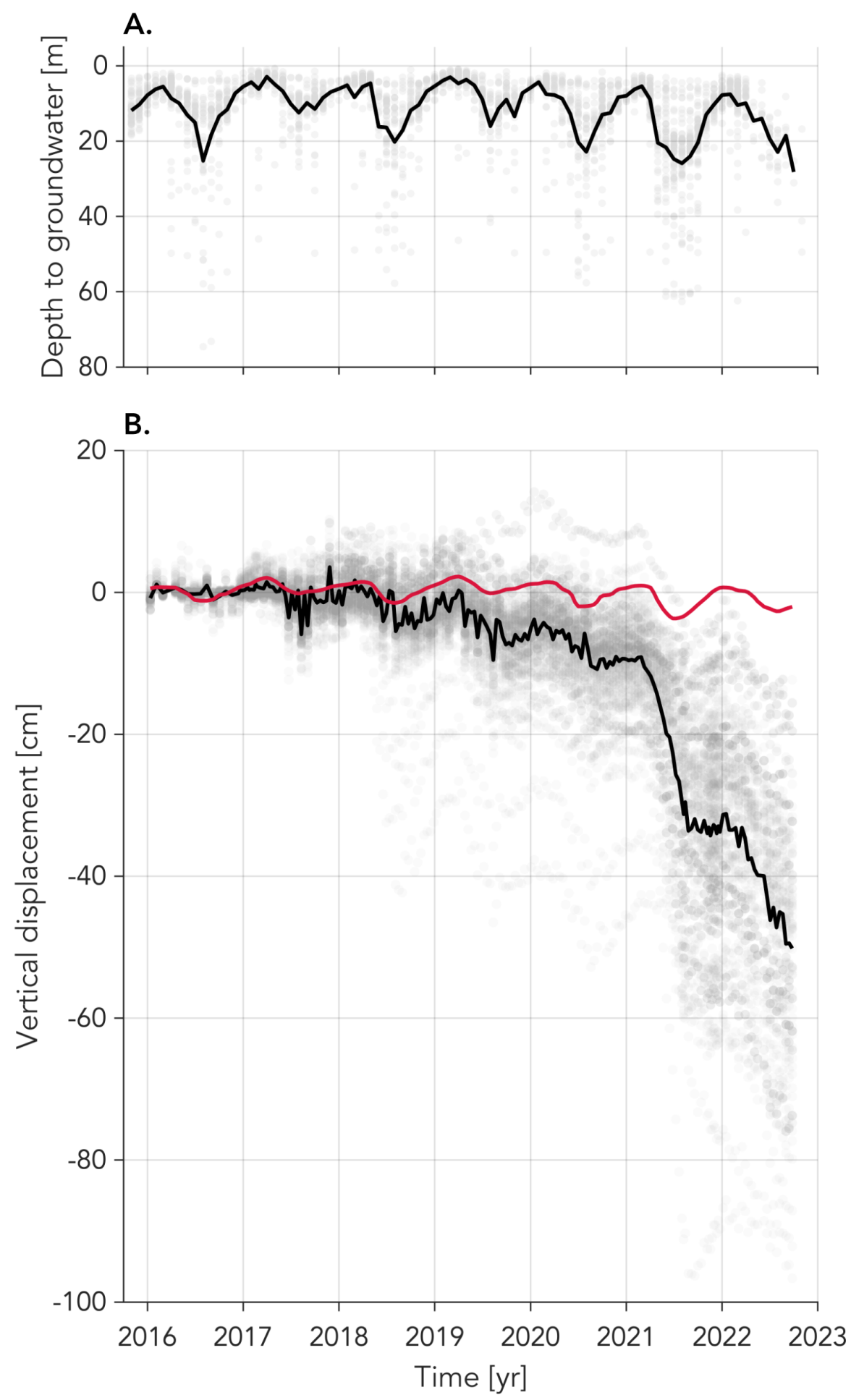

Fig. S3.8. (A) Stacked (grey points) and mean groundwater levels (black line) for the 96 wells located in zones with residual subsidence rates greater than 15 cm/yr. (B) Corresponding stacked (grey points) and mean (black line) InSAR-measured displacements with the mean inferred poroelastic displacements (red line).

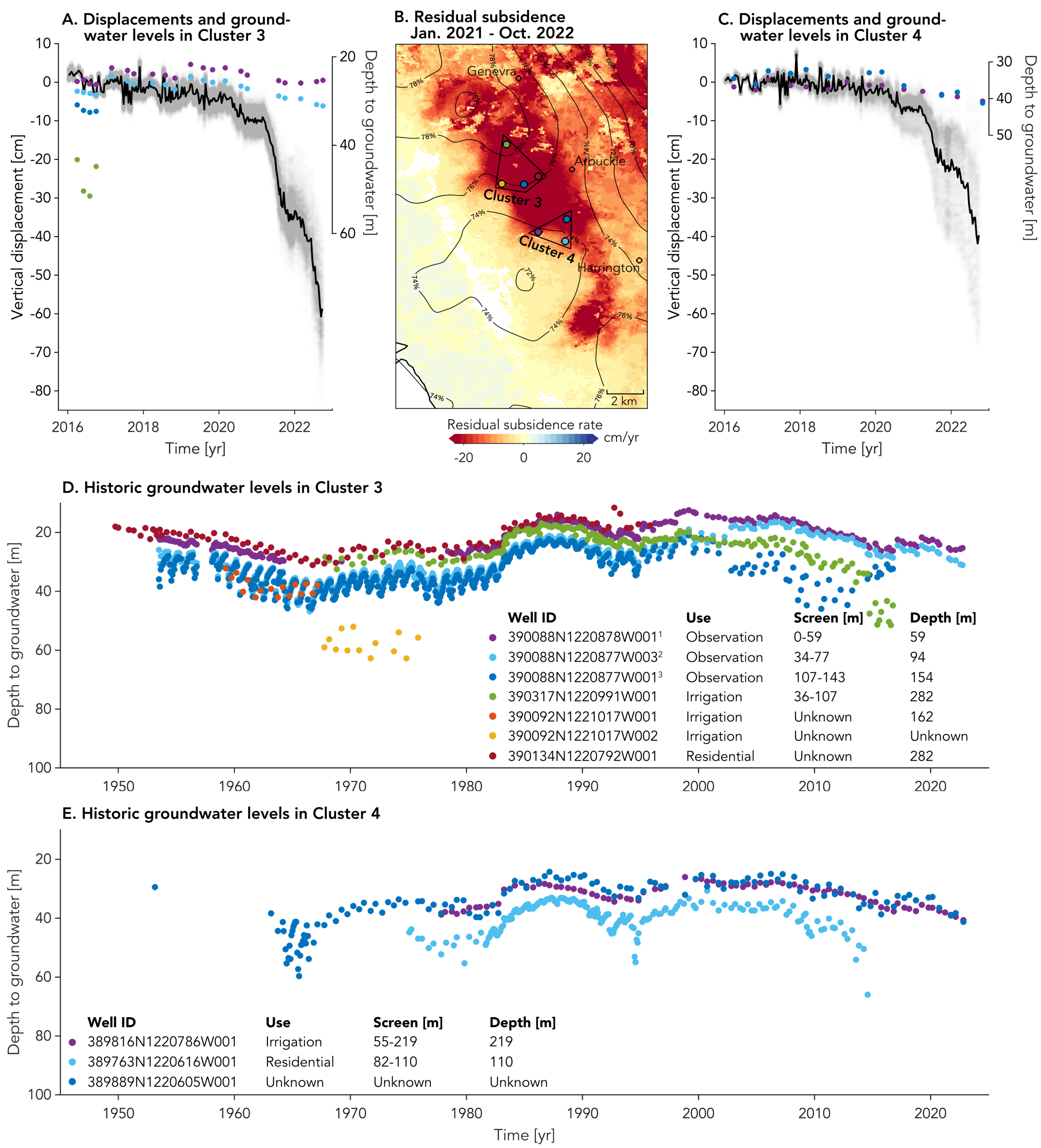

Station Comments:

[1] BE CAUTIOUS IF USED IN GWL CHANGE, CONTOUR OR DOT MAPS (casing collapsed & broke in adjacent monitoring well an TC canal buckled to North). This well is caved at ~ 90' depth.
[2] BE CAUTIOUS IF USED IN GWL CHANGE, CONTOUR OR DOT MAPS (Adjacent casing collapsed & broke): This well total depth was tagged at 187' on 7/2017.
[3] DO NOT USE IN GWL CHANGE, CONTOUR OR DOT MAPS (casing collapsed & broke) Cased to 470' - effective portion of well is from 330' to 505'.

Fig. S3.9. Similar to Fig. 4 but for well clusters close to Arbuckle (see Fig. 3E for location). We note that some of the well records in Cluster 3 may not be accurate as indicated by the station comments in the DWR database.

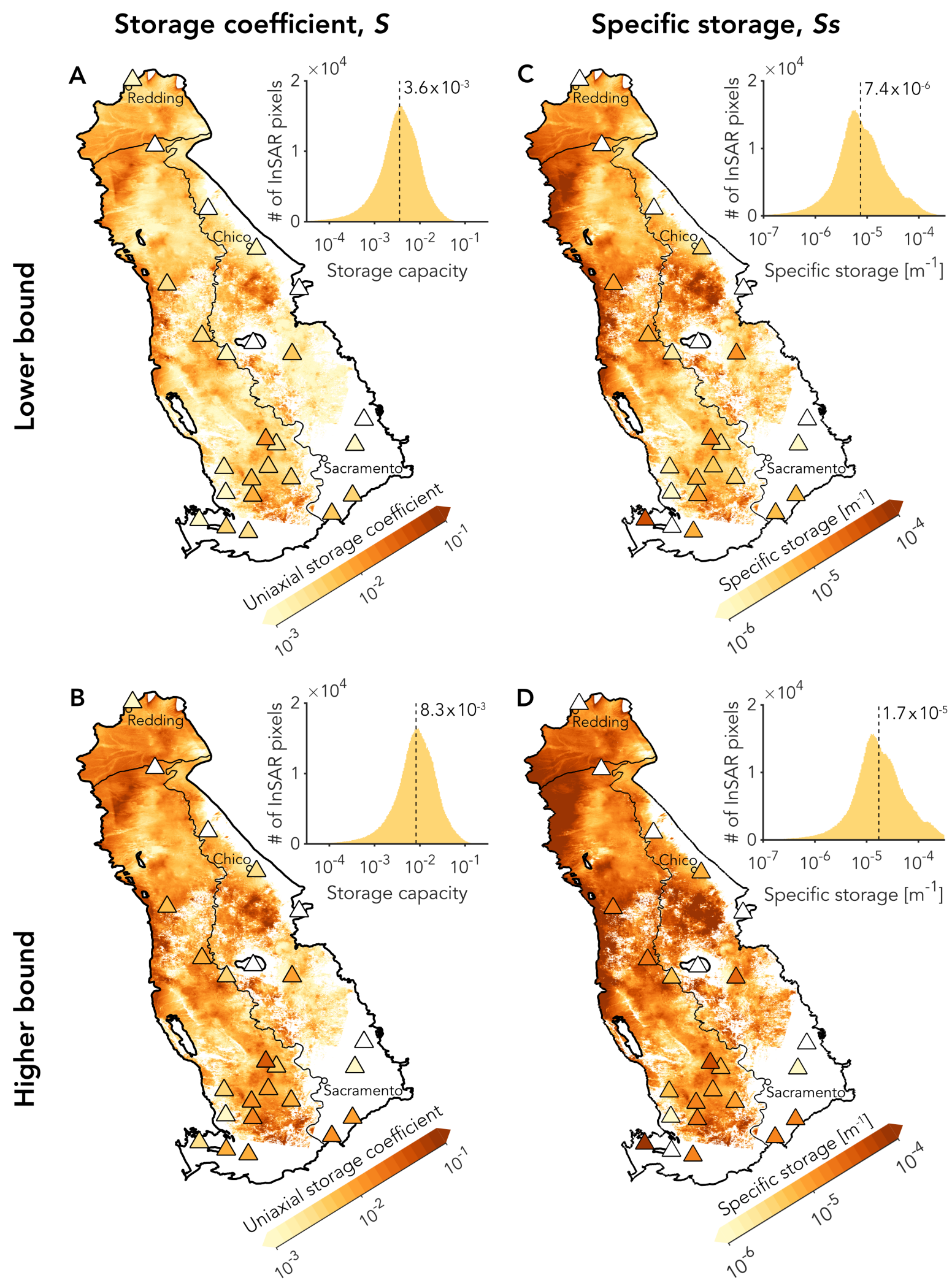

Fig. S4.1. Lower- (A,C) and upper-bound (B,D) distributions of inferred uniaxial storage coefficient (A,B) and uniaxial specific storage (C,D). The distributions are also shown in histogram format along with their logarithmic mean.
